# Mitophagy-associated biomarkers and macrophage involvement in pulmonary arterial hypertension: identification and functional implications

**DOI:** 10.3389/fphys.2025.1673181

**Published:** 2025-11-05

**Authors:** Xiaoyu Zhang, Liming Cheng, Jiahui Xie, Xuejuan Ma, Wenting Gui, Jiaxiang Chen, Kai Liu, Runwei Ma

**Affiliations:** ^1^ Department of Cardiac Surgery, Fuwai Yunnan Hospital, Chinese Academy of Medical Sciences/Affiliated Cardiovascular Hospital of Kunming Medical University, Kunming, Yunnan, China; ^2^ Department of Anesthesiology, Kunming Children’s Hospital, Kunming, Yunnan, China; ^3^ Department of Ultrasonography, The First Affiliated Hospital of Kunming Medical University, Kunming, Yunnan, China; ^4^ Department of Comprehensive Pediatrics, Kunming Children’s Hospital, Kunming, Yunnan, China

**Keywords:** biomarkers, macrophages, mitophagy, pulmonary arterial hypertension, single-cell RNA sequencing

## Abstract

**Background:**

Pulmonary arterial hypertension (PAH) is a progressive disorder characterized by pulmonary vascular remodeling and mitochondrial dysfunction. Recent studies have implicated impaired mitophagy in the pathogenesis of PAH; however, the underlying mechanisms and associated biomarkers remain insufficiently defined. This study used an integrative approach, incorporating bulk transcriptomic profiling, single-cell RNA sequencing (scRNA-seq), machine learning algorithms, and experimental validation to explore the relationship between mitophagy and PAH.

**Methods:**

Differentially expressed genes were extracted from publicly available microarray datasets and intersected with mitophagy-related genes curated from the MitoCarta 3.0 database. Weighted gene co-expression network analysis, along with five distinct machine learning models, identified five candidate mitophagy-associated biomarkers: *RRAS*, *BECN1*, *MFN1*, *HIF1A*, and *TAX1BP1*. These genes demonstrated high diagnostic performance (area under the curve >0.9) across both training and validation cohorts. Immune cell deconvolution analysis indicated a marked increase in M1 macrophage infiltration in lung tissue from individuals with PAH. The scRNA-seq further localized the expression of these biomarkers predominantly to monocyte/macrophage populations and indicated distinct pseudotemporal expression trajectories during macrophage differentiation. Expression and co-localization of the identified biomarkers with autophagy and inflammation markers were subsequently validated using quantitative PCR, western blotting, and immunofluorescence in a monocrotaline-induced PAH rat model.

**Results and Conclusion:**

The findings underscore the involvement of mitophagy in the pathobiology of PAH and identify five macrophage-associated biomarkers with strong diagnostic potential. These results may inform future strategies aimed at early detection and targeted therapeutic interventions in PAH.

## 1 Introduction

Pulmonary arterial hypertension (PAH) is a progressive disorder defined by sustained elevation in pulmonary arterial pressure, with key pathological features including vascular remodeling, inflammation, and right ventricular dysfunction (Thenappan et al., 2018). Despite recent advances in diagnostic and therapeutic modalities, the underlying pathophysiological mechanisms of PAH remain only partially elucidated (Humbert et al., 2023; Ghofrani et al., 2025). Current treatment options remain largely inadequate in reversing disease progression, resulting in persistently poor prognoses and elevated mortality rates (Yang et al., 2020). Consequently, elucidating the molecular mechanisms underlying PAH and identifying reliable biomarkers for early diagnosis and novel therapeutic targets are critical priorities in ongoing research.

Among the various mitochondrial quality control processes, mitophagy has emerged as a mechanism of growing interest. Mitophagy involves the selective encapsulation of damaged or dysfunctional mitochondria within autophagosomes, which fuse with lysosomes to facilitate degradation and recycling of mitochondrial components (Wang et al., 2023). This process is vital for preserving mitochondrial integrity and cellular homeostasis, particularly in response to oxidative stress or energy deficiency (Doblado et al., 2021). Aberrations in mitophagy whether excessive or insufficient have been associated with a range of pathological states, including cardiovascular disease, malignancy, and inflammatory conditions (Levine and Kroemer, 2019; Hoffmann et al., 2013; Yamamoto et al., 2023). Therefore, the exploration of mitophagy-related pathways and biomarkers in PAH may yield valuable insights into disease pathogenesis and support the development of targeted diagnostic and therapeutic strategies.

Emerging evidence indicates that the progression of pulmonary diseases is frequently associated with cell-type-specific dysregulation of mitophagy (Sharma et al., 2021). The rapid development of single-cell RNA sequencing (scRNA-seq) technologies has provided an advanced platform for dissecting cellular heterogeneity and characterizing intercellular interactions within complex disease environments. This technology enables the identification of distinct molecular profiles across diverse cell populations, allowing for the detailed investigation of key cellular contributors to the pathogenesis of PAH (Ziegenhain et al., 2017; Jovic et al., 2022).

In this study, PAH was examined through the integration of publicly available transcriptomic datasets, single-cell transcriptomics, machine learning methodologies, and experimental validation. This multi-modal approach was used to explore the involvement of mitophagy and monocyte/macrophage populations in disease progression, with the aim of elucidating underlying molecular mechanisms and identifying candidate diagnostic and therapeutic targets.

## 2 Materials and methods

### 2.1 Data sources

The scRNA-seq data (GSE210248) (Crnkovic et al., 2022) and microarray transcriptomic datasets (GSE113439) (Mura et al., 2019) and (GSE130391) (Fu et al., 2022) were obtained from the Gene Expression Omnibus (GEO) database (http://www.ncbi.nlm.nih.gov/geo/). The GSE210248 dataset, generated using the GPL20301 platform, consisted of six pulmonary artery samples, including three from patients diagnosed with PAH and three from healthy controls. The GSE113439 and GSE130391 datasets were generated based on the GPL6244 and GPL570 platforms, respectively.

For the present analysis, the GSE113439 dataset was used as the training set and included lung tissue samples from 15 individuals with PAH and 11 healthy controls (Supplementary Table S1). The GSE53408 dataset, consisting of 12 PAH lung tissue samples and 11 normal lung tissue samples, was used as the validation set in subsequent analyses.

A total of 65 mitophagy-related genes (MRGs) (SupplementaryTable S2) were retrieved from the MitoCarta 3.0 database (https://www.broadinstitute.org/mitocarta). A waiver for ethics approval for the human public database component of the study has been obtained.

### 2.2 Differential expression analysis

Differentially expressed genes (DEGs) between PAH and control samples in the training set were identified using the “limma” package (version 3.57.11), with threshold criteria set at |log_2_ fold change (FC)| >0.5, adj.P.Val <0.05 (Liu et al., 2021). A volcano plot was generated using the “ggVolcano” package (version 0.0.2) to visualize the distribution of all DEGs (Simon et al., 2011). Additionally, a heatmap was constructed with the “ComplexHeatmap” package (version 2.17.0) to depict the expression patterns of DEGs and their associations with mitochondrial autophagy-related pathways.

### 2.3 Establishment of the PAH animal model

Twelve male Sprague–Dawley (SD) rats (6–8 weeks old) were procured from Beijing Sipai Fu Laboratory Animal Co., Ltd (Production License No. SCXK [Beijing] 2019–0010; Use License No. SYXK [Yunnan] K2022-0007) and maintained under specific pathogen-free conditions. After 3 days of adaptive feeding, the subjects were randomly divided into a control group (n = 6) and a PAH group (n = 6). The PAH group was administered a single intraperitoneal injection of monocrotaline (MCT; 60 mg/kg, Sigma-Aldrich, St. Louis, MO), in accordance with established protocols (Zhai et al., 2022), while the control group received an equivalent volume of sterile saline (Liangshuiting, Lot No. L24041109). Fourteen days following injection, all animals were anaesthetized with sodium pentobarbital (50 mg/kg, intraperitoneally, Lot No. M50179, Cas No. 57–33–0, Shanghai FWD Chemicals Limited) for echocardiographic assessment. At the end of the experiment, euthanasia was performed with sodium pentobarbital (100–200 mg/kg, intraperitoneally, Lot No. M50179, Cas No. 57–33–0, Shanghai FWD Chemicals Limited). Lung tissues were subsequently harvested for subsequent analyses.

### 2.4 Echocardiographic assessment

Fourteen days following subcutaneous administration of monocrotaline or saline (as described in Section 2.3), SD rats were anaesthetized with pentobarbital sodium (50 mg/kg, intraperitoneally). The anterior thoracic region was depilated, and each animal was positioned in a supine orientation and immobilized. Pulmonary resistance was assessed using a cardiac color Doppler ultrasound system (Mindray, M9 Ultrasound System, SP5-1s probe, Shenzhen, Guangdong, China). Sampling was performed at the pulmonary artery in the short-axis view of the great arteries. Pulmonary artery acceleration time (PAAT) and pulmonary artery ejection time (PAET) were measured upon acquisition of the systolic blood flow spectrum of the pulmonary artery. The ratio of PAAT to PAET (PAAT/PAET) was subsequently calculated (Trittmann et al., 2022).

### 2.5 Hematoxylin and eosin (H&E) staining

Paraffin-embedded lung tissue blocks were sectioned at a thickness of 5 μm. The sections were mounted on glass slides and baked in a 64 °C oven (Tianjin Borry Instrument Equipment Co., Ltd., GFL-230) for 1 h. Deparaffinization was performed using xylene (SCRC, 10023418), followed by rehydration through a graded ethanol series (100%, 95%, 80%, 70%; SCRC, 100092683), and rinsing in distilled water. The sections were stained with hematoxylin (Servicebio, G1076) for 5 min, the slides were counterstained with Eosin Y (Servicebio, G1076) for 2 min. Subsequently, it dehydrated and became transparent. The sections were then mounted using neutral gum (SCRC, 10004160). Representative microscopic fields were imaged under a light microscope.

### 2.6 Immunofluorescence

Fresh lung tissue sections (5 μm) were fixed in 4% paraformaldehyde (Servicebio, G1101) for 30 min at room temperature and subsequently permeabilized with 0.2% Triton X-100 in PBS for 15 min. The sections were blocked with PBS containing 5% bovine serum albumin (BSA) and 2% normal goat serum for 1 h at room temperature. Incubation with a primary antibody against light chain 3 (LC3) (Servicebio, GB11124; 1:2000) was carried out overnight at 4 °C. After three PBS washes, the sections were incubated with an HRP-conjugated goat anti-rabbit/mouse IgG secondary antibody (Servicebio; 1:500) for 1 h at 37 °C. Following final washes, slides were mounted using an antifade medium and examined under a fluorescence microscope.

### 2.7 Transmission electron microscopy (TEM)

Lung tissue fixed in electron microscopy fixative (Servicebio, G1102) at 4 °C for 2–4 h and embedded in pre-warmed 1% agarose (Thermo Fisher, 16520100). Post-fixation was conducted in 1% osmium tetroxide (Ted Pella, 18456) prepared in 0.1 M phosphate buffer (PB, pH 7.4) for 2 h at room temperature in the dark.

The samples were rinsed with phosphate buffer and dehydrated using a graded acetone series (Xilong Scientific, 230106), followed by infiltration with increasing concentrations of 812 resin (SPI, 90529-77-4) and polymerization at 60 °C for 48 h. Ultrathin sections (60–80 nm) were prepared using an ultramicrotome (Leica RM2135) and mounted on 150-mesh formvar-coated copper grids. Sections were stained with 2% uranyl acetate (SPI, 02624-AB) for 8 min in the dark, washed with 70% ethanol and ultrapure water, and subsequently stained with 2.6% lead citrate (Sigma, 203580) for 8 min while protected from CO_2_ exposure. After final washes, the grids were air-dried overnight and examined using a TEM (JEOL JEM-1400 Flash, JEOL Ltd., Tokyo, Japan) for imaging.

### 2.8 WGCNA

To identify gene modules most strongly associated with the PAH phenotype, weighted gene co-expression network analysis (WGCNA) was conducted on all samples in the training set using the WGCNA package (version 1.72-5) (Langfelder and Horvath, 2008). An unsigned network was constructed based on a dissimilarity measure derived from the topological overlap matrix, and hierarchical clustering of genes was performed using the average linkage method. Modules were defined through the application of a dynamic tree-cut algorithm, and correlations between module eigengenes and the PAH phenotype were assessed. Modules with an absolute correlation value (|correlation|) greater than 0.3 and a p-value less than 0.05 were considered statistically significant. Select the appropriate soft threshold power from modules one to 14, set R2 = 0.85, and filter the soft threshold β value to 10, minModuleSize = 100, mergeCutHeight = 0.15.

For each gene, module membership (MM), defined as the correlation with the corresponding module eigengene, and gene significance (GS), defined as the correlation with PAH status, were calculated. Genes within key modules satisfying MM >0.8 and GS >0.2 were selected for downstream analyses.

### 2.9 Identification and functional analysis of candidate genes

To identify candidate biomarkers associated with PAH, the DEGs, key module genes obtained from WGCNA, and MRGs were intersected using the ComplexUpset package (version 1.3.3) (Lex et al., 2014). Functional enrichment analysis of the intersected gene set was conducted using the clusterProfiler package (version 4.9.4) (Wu et al., 2021) for Gene Ontology (GO) terms and Kyoto Encyclopedia of Genes and Genomes (KEGG) pathways, applying a significance threshold of p < 0.05. A protein-protein interaction (PPI) network was constructed through the STRING database (https://cn.string-db.org/) and visualized using Cytoscape, with the minimum interaction score set to 0.15.

### 2.10 Machine learning–based biomarker selection

To further refine the list of DEGs associated with PAH and mitophagy, five machine learning algorithms support vector machine (SVM), generalized linear model (GLM), neural network (NN), random forest (RF), and extreme gradient boosting (XGBoost) were applied to the training set using the caret package (version 6.0–94). Model interpretability and variable importance were assessed using the DALEX package (version 2.4.3) (Guan et al., 2023). Candidate feature genes were subsequently validated in the independent validation set, with statistical significance defined as *p* < 0.05. Receiver operating characteristic (ROC) curves and area under the curve (AUC) values were computed using the *pROC* package (version 1.18.4) (Robin et al., 2011), and an AUC greater than 0.7 was considered indicative of satisfactory predictive performance. Final biomarkers were selected based on a combination of gene expression profiles and ROC curve analysis.

### 2.11 Functional annotation of biomarkers

Spearman’s correlation analysis was performed between each identified biomarker and all other genes in the training set. Genes were ranked according to the strength of correlation and subjected to gene set enrichment analysis (GSEA) using the “c2. kegg.symbols.gmt” collection from the Molecular Signatures Database (MSigDB) (https://www.gsea-msigdb.org/gsea/msigdb), applying a significance threshold of *p* < 0.05. Circos plots depicting the chromosomal distribution of the validated biomarkers were generated using the Circos package (version 0.69) (Krzywinski et al., 2009).

### 2.12 Immune cell infiltration analysis

The relative proportions of 22 immune cell types in PAH and control samples from the GSE113439 training set were estimated using the CIBERSORT algorithm (version 1.03) (Newman et al., 2015). Group differences were assessed using the Wilcoxon rank-sum test, and correlations between infiltrating immune cell types and identified biomarkers were assessed using Spearman’s correlation method (|correlation| >0.3, *p* < 0.05). The results were visualized with the ggplot2 package (version 3.4.2) (Gustavsson et al., 2022).

### 2.13 Single‐cell RNA‐seq data analysis

#### 2.13.1 Quality control

Raw counts from the GSE210248 dataset were processed using the Seurat package (version 4.1.1) (Satija et al., 2015). Cells were retained based on the criteria of expressing more than 200 genes, containing fewer than 10,000 unique molecular identifier counts, and exhibiting mitochondrial gene content below 5%. Gene expression matrices were normalized and scaled using the LogNormalize method. Highly variable genes (HVGs) were identified using the FindVariableFeatures function with the “vst” selection method, and the top 2,000 HVGs were selected for downstream analyses.

#### 2.13.2 Cell clustering and annotation

Principal component analysis was conducted on the scaled data, and the significance of principal components (PCs) was assessed using the JackStraw and ScoreJackStraw functions. Dimensionality reduction and visualization were performed using Uniform Manifold Approximation and Projection (UMAP). Clustering was carried out as a resolution selected to optimize separation, with statistical significance defined as *p* < 0.05. Cell type annotation was conducted by cross-referencing canonical marker genes reported in the literature (Crnkovic et al., 2022).

#### 2.13.3 Cell–cell communication analysis

Intercellular signaling networks were inferred using the CellChat package in conjunction with the CellChatDB.human reference database. Predicted ligand–receptor interactions and enriched signaling pathways between annotated cell types were analyzed to elucidate potential modes of cellular communication.

### 2.14 Gene set variation analysis (GSVA) and pseudotime trajectory of key cells

GSVA scores for each cell were calculated using the GSVA package (version 1.49.8) and applied to the validated biomarker gene set. Differences in biomarker GSVA scores between PAH and control samples across cell clusters were assessed using the Wilcoxon rank-sum test, with statistical significance defined as *p* < 0.05 (Hänzelmann et al., 2013). To identify key cell populations, Hallmark pathway GSVA scores were computed for each cell subcluster using the “h.all.v2022.1. Hs.symbols.gmt” gene set. Pathways demonstrating significant differences based on the Kruskal–Wallis test (*p* < 0.05) were visualized in a heatmap. Within the identified key cluster, additional sub-clustering was performed at a resolution of 0.02 to delineate cellular heterogeneity. Pseudotemporal ordering was reconstructed using Monocle2, based on genes with high variability (q < 0.1), and dimensionality reduction was performed using the DDRTree algorithm to infer developmental trajectories and order cells accordingly (Ionkina et al., 2021).

### 2.15 qPCR

Total RNA was extracted from lung tissue using TRIzol reagent (Ambion), following the manufacturer’s protocol. After measuring the concentration, reverse transcribe mRNA into cDNA. Quantitative PCR (qPCR) was conducted using the EasyPure® qPCR SuperMix Kit (TransGen Biotech, China, Cat. No. ER101-01) with gene-specific primers (Table 1). Relative gene expression levels were determined using the 2^−ΔΔCt^ method, with GAPDH serving as the internal control.

**TABLE 1:** Primer sequence.

Gene	Sequence 5’-3’
RRAS-F	GTCCTCAGCCCGACATCTCA
RRAS-R	GCTGGTCACTTGAGGCTACA
BECN1-F	CTTCAATGCGACCTTCCA
BECN1-R	TACAACGGCAACTCCTTAG
MFN1-F	GCAGCACCAGATAATGCAGC
MFN1-R	GCTCTGGTGGAGAAACTGCT
HIF1A-F	AAGCAGCAGGAATTGGAACG
HIF1A-R	CGTAACTGGTCAGCTGTGGT
TAX1BP1-F	TGGATGTAAAGCCAGCAGCA
TAX1BP1-R	GCACCATCTGCTCCATCTCT
GAPDH-F	AGTCTACTGGCGTCTTCACC
GAPDH-R	CCACGATGCCAAAGTTGTCA

### 2.16 WB

Lung tissue samples were homogenized in 500 µL of RIPA lysis buffer (Servicebio, G2002-30 mL) supplemented with protease inhibitor cocktail (Proteintech, PR20032) on ice for 10 min and subsequently centrifuged at 14,000 × g for 15 min at 4 °C. Protein concentrations in the supernatant were determined using a BCA assay kit (Merck, BCA1-1KT). Aliquots containing 80 µg of protein were mixed with 20 µL of 5× loading buffer, boiled for 5 min, and separated on 10% SDS–PAGE gels (Solarbio, G2017).

Proteins were transferred onto PVDF membranes (Merck Millipore, SEQ00010) and blocked with 5% nonfat milk in TBS-T for 1 h at room temperature. Membranes were incubated overnight at 4 °C with primary antibodies diluted in blocking buffer: RRAS (Proteintech, 66959-1-Ig; 1:1000), BECN1 (Proteintech, 66665-1-Ig; 1:1000), MFN1 (Zenbio, R27027; 1:1000), TAX1BP1 (Hanan Biotechnology, HA721648; 1:1000), HIF1A (Abcam, ab179483; 1:1000), and β-actin (Proteintech, 66009-1-Ig; 1:25,000). After washing, membranes were incubated for 40 min at room temperature with either HRP-conjugated goat anti-rabbit IgG (Proteintech, SA00001-2; 1:3000) or HRP-conjugated goat anti-mouse IgG (Servicebio, GB23301; 1:3000).

Protein bands were visualized using Immobilon® UltraPlus Western HRP substrate (Millipore, WBULS0500) and imaged using the Bio-Rad ChemiDoc™ XRS + system (Bio-Rad, 1708265). Densitometric analysis was conducted in ImageJ (v1.8.0.345), and the expression levels of target proteins were normalized to β-actin (relative quantification = band gray valuetarget ÷ band gray valueβ-actin).

### 2.17 Dual immunofluorescence staining

Lung tissue slices (5 μ m) were fixed overnight with 4% paraformaldehyde (Servicebio, G1101) at room temperature. Following permeabilization with 0.2% Triton X-100 in PBS, the sections were blocked with PBS containing 5% normal goat serum and 2% BSA for 1 h at room temperature. Incubation with primary antibodies against inducible nitric oxide synthase (iNOS) (Servicebio, GB11119; 1:1000) and LC3 (Servicebio, GB11124; 1:2000), diluted in blocking buffer, was carried out overnight at 4 °C. After washing, the sections were incubated for 20 min at 37 °C with HRP-conjugated goat anti-rabbit IgG (Servicebio, GB23303; 1:500) and HRP-conjugated goat anti-mouse IgG (Servicebio, GB23301; 1:500).

Nuclei were counterstained with DAPI (Servicebio, G1012) for 7 min at room temperature. Slides were mounted using antifade medium (Servicebio, G1401). Five fields per section: one central and four peripherals were imaged using a fluorescence microscope. Fluorescence intensity and the rate of positively stained cells were quantified using Image-Pro Plus software.

### 2.18 Statistical analysis

All statistical analyses were conducted using R software (version 4.1.3). Data are presented as mean ± standard deviation. Comparisons between two groups were conducted using either a two-tailed Student’s t-test or the Wilcoxon rank-sum test, as appropriate. *p* < 0.05 was considered statistically significant.

## 3 Results

### 3.1 Bioinformatics analysis and *in vivo* validation of the relationship between PAH and mitophagy

**FIGURE 1:**
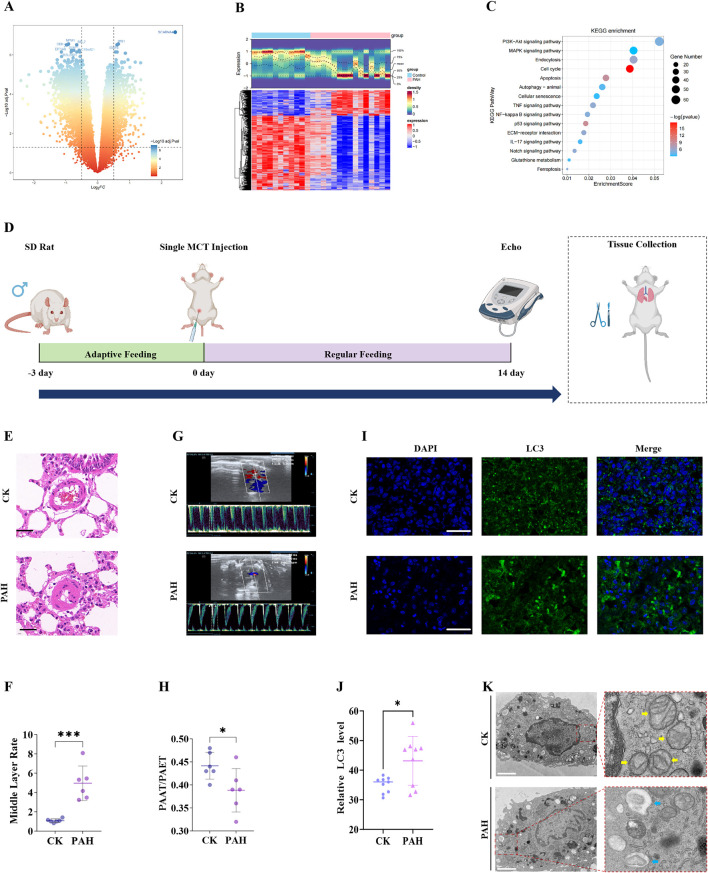
Bioinformatics analysis of PAH‐associated DEGs and *in vivo* validation. **(A)** volcano plot of DEGs in the GSE113439 training set; **(B)** Heatmap of DEGs; **(C)** KEGG pathway enrichment analysis shows a significant association of DEGs with mitophagy‐related pathways; **(D)** Schematic of the monocrotaline‐induced PAH rat model; **(E)** H&E staining of rat lung tissue demonstrating histopathological changes (Scale bar 50 μm); **(F)** quantification of pulmonary arterial medial wall thickness from H&E images. **(G)** Echocardiographic assessment of PAH model validation. **(H)** quantitative echocardiographic parameters: PAAT/PAET; **(I)** Immunofluorescence detection of LC3 expression in rat lung tissue (Scale bar 100 μm); **(J)** quantitative analysis of LC3 immunofluorescence intensity; **(K)** TEM of mitochondrial morphology: yellow arrows indicate normal mitochondria in the CK,and blue arrows indicate mitophagosome formation and reduced mitochondrial area in the PAH group. Scale bar: 2 μm. Data are presented as mean ± SEM. ns, p > 0.05; *p < 0.05; **p < 0.01; ***p < 0.001; ****p < 0.0001.

To explore the relationship between PAH and mitochondrial dynamics, the GSE113439 training set was analyzed, and 2,753 DEGs associated with PAH, including 698 upregulated and 2,055 downregulated genes (Figures 1A,B; Supplementary Table S3) were identified. KEGG pathway enrichment analysis indicated that these DEGs were significantly involved in mitophagy-related pathways (Figure 1C). A rat model of PAH was subsequently established through a single subcutaneous injection of monocrotaline (60 mg/kg) at the nape (Figure 1D).

H&E staining indicated a significant increase in medial wall thickness of pulmonary arterioles in the PAH group compared to the control group (Figures 1E,F; Supplementary Table S4). Consistent with histological findings, echocardiographic assessment demonstrated significantly elevated distal pulmonary vascular resistance in PAH rats compared to controls (Figures 1G,H; Supplementary Table S5), thereby confirming successful model induction. Immunofluorescence staining presented a pronounced upregulation of the autophagy marker LC3 in the PAH group, indicative of increased autophagosome formation under PAH conditions (Figures 1I,J; Supplementary Table S6). TEM further demonstrated prominent mitochondrial structural abnormalities in PAH lung tissue, including the formation of mitophagosomes (Figure 1K), corroborating the LC3 immunofluorescence results.

These findings collectively indicate that PAH is associated with mitophagosome formation and disruption of mitochondrial integrity. However, further investigation is required to identify the specific PAH-associated genes contributing to mitochondrial dysfunction.

### 3.2 Identification and analysis of candidate genes

**FIGURE 2:**
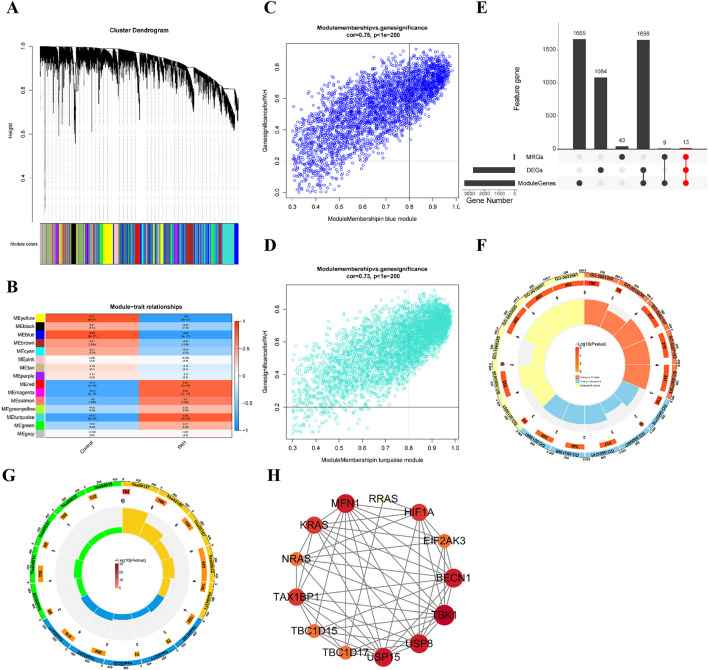
Identification and analysis of 13 candidate genes associated with PAH and mitophagy. **(A)** dendrogram showing the 14 co‐expression modules identified by WGCNA; **(B)** Heatmap of module-trait correlations: The blue and turquoise modules exhibit the strongest correlations with PAH status; **(C)** scatterplot of gene significance *versus* module membership for the blue model; **(D)** Scatterplot of gene significance *versus* module membership for the turquoise model; **(E)** venn diagram illustrating the intersection of DEGs, key module genes, and MRGs, yielding 13 candidates; **(F)** GO enrichment analysis of the 13 candidate genes, showing the top enriched term; **(G)** KEGG pathway enrichment bubble chart for the 13 candidate genes; **(H)** PPI network of the 13 candidate genes.

Integrated bioinformatics analyses and *in vivo* validation indicated a key role for mitophagy in the pathogenesis of PAH. WGCNA was conducted on all samples in the training set (Supplementary Table S7), with no outlier samples found (Supplementary Figure S1A). A total of 14 co-expression modules were identified (Figure 2A; Supplementary Figure S1B). Among these, the blue module (module eigengene correlation with PAH status: r = −0.81) and the turquoise module (r = 0.76) were identified as key modules (Figure 2B). Together, these two modules encompassed 3,343 genes, including 1,086 in the blue module (Figure 2C) and 2,257 in the turquoise module (Figure 2D).

A total of 65 MRGs were retrieved from the MitoCarta 3.0 database. The intersection of DEGs, key module genes, and MRGs resulted in the identification of 13 candidate genes (Figure 2E; Supplementary Table S8). GO and KEGG pathway enrichment analyses were performed to explore their functional relevance. GO analysis indicated 58 significantly enriched terms, comprising 27 biological process, seven molecular function, and 24 cellular component categories (Supplementary Table S9). The top five enriched terms included GDP binding, GTPase activity, K48-linked deubiquitinase activity, GTP binding, and guanyl nucleotide binding (Figure 2F). KEGG pathway analysis identified 70 enriched pathways, including mitophagy animal, autophagy animal, kaposi’s sarcoma associated herpesvirus infection, neurodegenerative disease multiple diseases, and opioid peptide signaling pathways (Figure 2G; Supplementary Table S9).

A PPI network was constructed using the STRING database, comprising of 13 nodes and 54 edges (Figure 2H). Within this network, *RRAS*, *BECN1*, *MFN1*, *HIF1A*, and *TAX1BP1* presented the highest number of interactions. Overall, the results of WGCNA and intersection analyses supported the central involvement of mitophagy in PAH and identified 13 candidate genes implicated in diverse biological pathways, particularly those related to mitochondrial autophagy.

### 3.3 Identifying biomarkers

**FIGURE 3:**
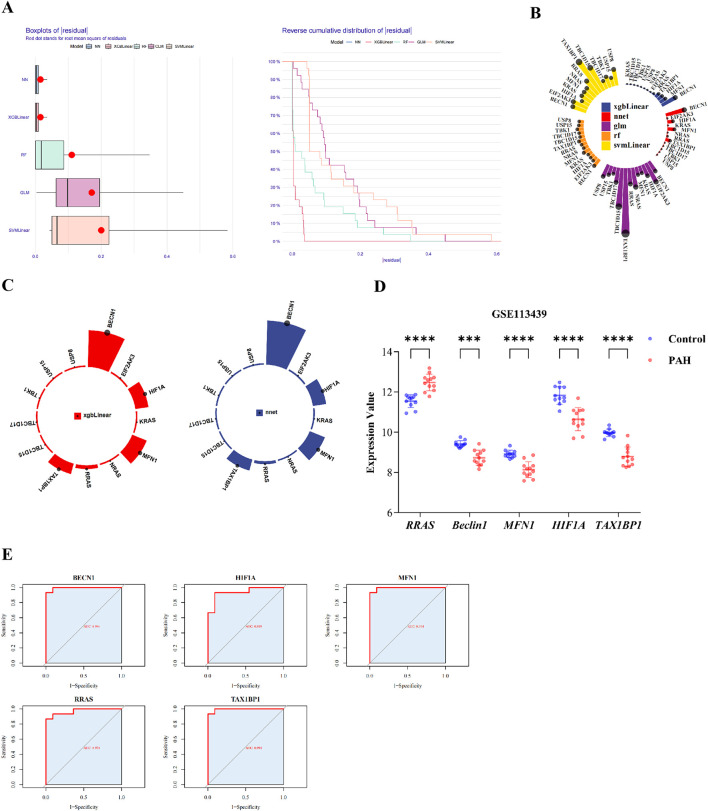
Identification of biomarkers and ROC analyses. **(A)** cumulative residual distribution plot; **(B)** Ranking of feature importance for candidate genes across five machine learning model; **(C)** feature importance ranking in the XGBLinear and neural network (NNet) model; **(D)** validation of biomarker expression in the training datasets; **(E)** ROC curves for each biomarker in the training set. Data are presented as mean ± SEM. ns, p > 0.05; *p < 0.05; **p < 0.01; ***p < 0.001; ****p < 0.0001.

To further refine biomarkers associated with mitochondrial autophagy and PAH, five machine learning models SVM, GLM, NN, RF, and XGBoost were trained using the training dataset. Model performance was subsequently assessed. Feature importance rankings derived from the XGBoost and neural network models were intersected, resulting in the identification of five top candidate genes (Figure 3A; Supplementary Table S10). These genes were designated as key biomarkers: *RRAS*, *BECN1*, *MFN1*, *HIF1A*, and *TAX1BP1* (Figures 3B,C).

The expression profiles of these five genes were assessed across both the training set (GSE113439) and the validation set (GSE53408). All five biomarkers demonstrated consistent patterns of differential expression (Figure 3D; Supplementary Figure S1E; Supplementary Table S11), with *BECN1*, *HIF1A*, *MFN1*, and *TAX1BP1* significantly downregulated in PAH samples, while *RRAS* was upregulated. ROC curve analysis indicated excellent diagnostic performance for each biomarker, with AUC values exceeding 0.90 in both datasets (Figure 3E; Supplementary Figure S1F; Supplementary Table S12).

From the integrated analyses of differential gene expression and diagnostic performance, *RRAS*, *BECN1*, *MFN1*, *HIF1A*, and *TAX1BP1* were identified as robust biomarkers associated with mitophagy dysregulation in PAH.

### 3.4 GSEA and immune infiltration of biomarkers

**FIGURE 4:**
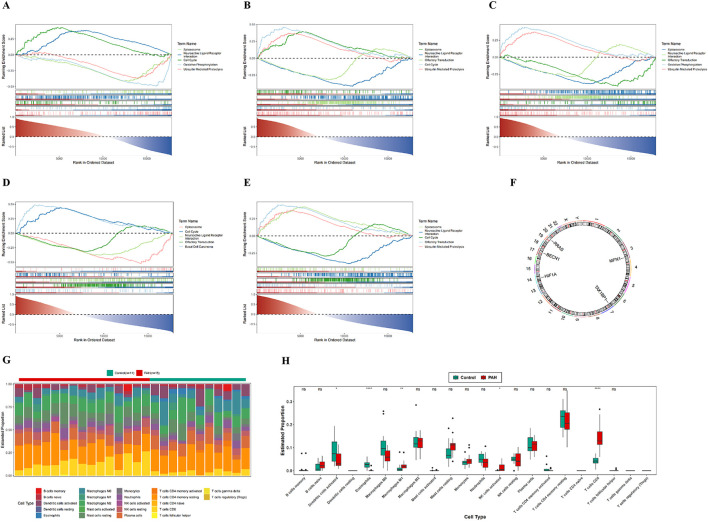
GSEA enrichment analysis, chromosomal localization, and immune infiltration analysis. **(A–E)** GSEA enrichment analysis of five candidate genes; **(F)** chromosomal localization map of five candidate genes; **(G)** infiltration analysis of 22 immune cell types between PAH patients and normal controls; **(H)** box plots of estimated proportions of 22 immune cell types in control and PAH lung tissues. In the figures, ns indicates p > 0.05; *p < 0.05; **p < 0.01; ***p < 0.001; ****p < 0.0001.

GSEA was conducted to elucidate the biological pathways associated with the identified biomarkers during the progression of PAH. *RRAS*, *BECN1*, *HIF1A*, and *TAX1BP1* were most significantly enriched in the spliceosome pathway, while *MFN1* presented the strongest enrichment in the neuroactive ligand–receptor interaction pathway. Notably, all five biomarkers exhibited co-enrichment in both the spliceosome and neuroactive ligand–receptor interaction pathways (Figures 4A–E; Supplementary Table S13).

Chromosomal mapping of the biomarkers indicated distinct genomic loci: *RRAS* on chromosome 19, *BECN1* on chromosome 17, *HIF1A* on chromosome 14, *TAX1BP1* on chromosome 7, and *MFN1* on chromosome 3 (Figure 4F; Supplementary Table S14). Immune cell infiltration in PAH *versus* control lung tissues was subsequently assessed using the CIBERSORT algorithm. Among the 22 immune cell subsets analyzed (Figure 4G; Supplementary Table S15), 5 cell types: M1 macrophages, eosinophils, activated natural killer (NK) cells, activated dendritic cells, and CD8^+^ T cells were found to differ significantly between the two groups (Figure 4H; Supplementary Table S15).

Correlation analysis demonstrated a positive association between CD8^+^ T cells and activated NK cells, and a negative association between CD8^+^ T cells and eosinophils (Supplementary Figure S1C; Supplementary Table S15). These findings indicate that, along with their distinct chromosomal distribution, the identified biomarkers are associated with specific alterations in the immune landscape of PAH. The elevated infiltration of M1 macrophages, eosinophils, activated NK cells, activated dendritic cells, and CD8^+^ T cells in PAH lung tissue indicates a potentially important role for these immune populations in the pathogenesis of the disease.

### 3.5 Functional characterization of five key biomarkers in macrophages revealed by scRNA‐Seq

To identify key cell populations associated with PAH, scRNA-seq data from GSE210248 were analyzed. Following quality control retaining cells with 200 to 10,000 found genes and fewer than 5% mitochondrial reads, a total of 21,794 cells were included in the analysis (Supplementary Figure S2A,B). Highly variable genes, including *SEPTIC*, *TPSAB1*, *APOE*, *S100A8*, *ACKR1*, *S100A9*, *CCL20*, *ACTA2*, and *LUM*, were identified and annotated (Supplementary Figure S2C). The top 30 PCs were visualized and assessed for statistical significance (Supplementary Figure S2D-F), and the top 10 marker genes per cluster were displayed in a heatmap (Supplementary Figure S2G).

**FIGURE 5:**
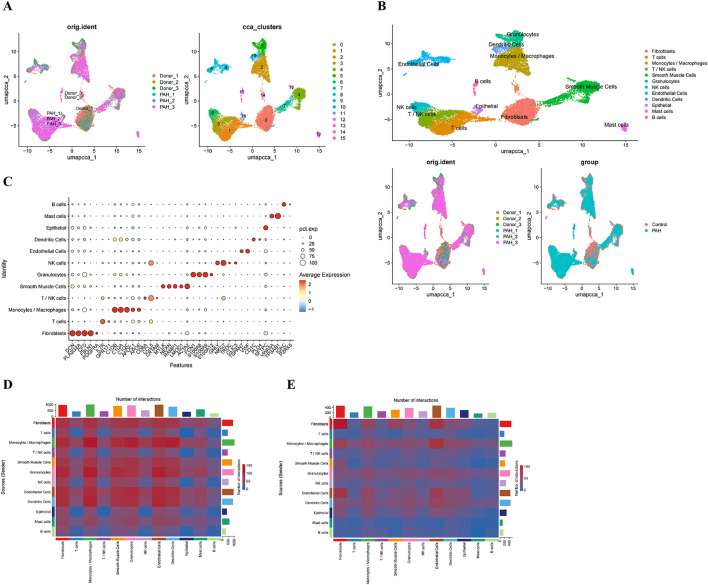
Single‐cell clustering and intercellular communication analyses. **(A)** UMAP projection of all cells prior to annotation; **(B)** UMAP plot with cells colored by annotated cell type; **(C)** expression of canonical marker genes across the annotated cell types; **(D, E)** Heatmaps showing the number of potential ligand-receptor pairs between cell types in the CK **(D)** and PAH **(E)**.

UMAP of the PCs segregated the cells into 16 distinct subclusters (Figure 5A). According to canonical marker genes from the literature, 12 cell types were annotated: B cells, mast cells, epithelial cells, dendritic cells, endothelial cells, NK cells, granulocytes, smooth muscle cells, T/NK cells, monocytes/macrophages, T cells, and fibroblasts (Figure 5B). Marker gene specificity analysis confirmed that each cell type expressed its respective canonical markers at the highest levels (Figure 5C).

Intercellular communication networks in control and PAH samples were inferred using the CellChat package (Figures 5D,E). T cells and T/NK cells were identified as major signal receivers in both groups, with the overall signaling strength observed to be greater in PAH. In contrast, monocytes/macrophages emerged as the dominant signal-sending population in PAH samples (Supplementary Figure S3A-D). Key signaling pathways mediating interactions involving T and T/NK cells included CCL, CXCL, MIF, galectin, IL-2, and SPP1 networks (Supplementary Figure S3E), whereas up to 12 major pathways were implicated in monocyte/macrophage-mediated interactions (Supplementary Figure S3F).

**FIGURE 6:**
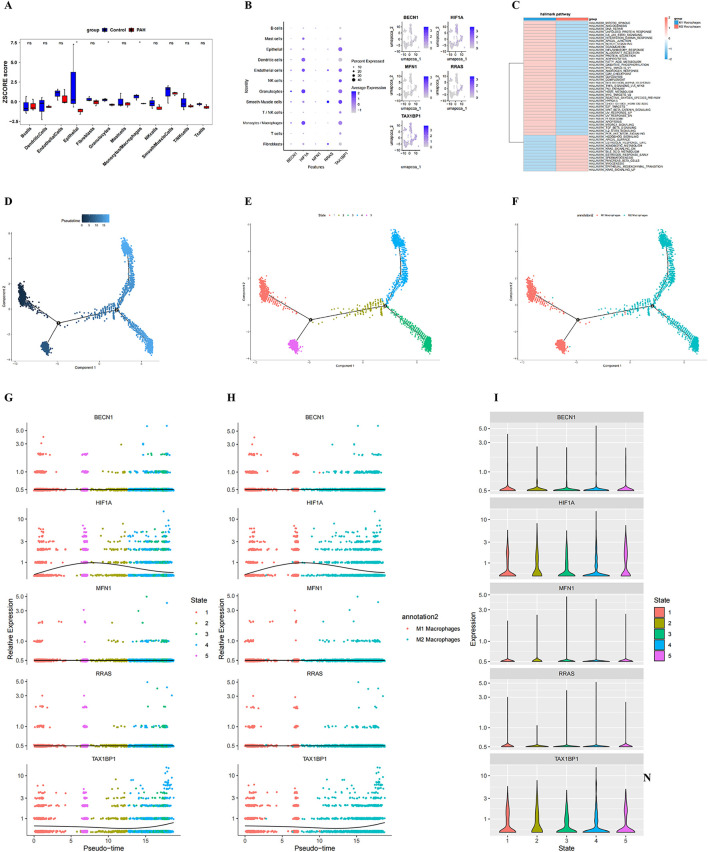
Identification of key cell type, pseudotime trajectories, and dynamic biomarker expression. **(A)** bar plot showing differential proportions of cell clusters between PAH and control samples; **(B)** Bubble plot illustrating the expression levels of the five candidate genes across annotated cell types; bubble size indicates the percentage of expressing cells, and colour intensity reflects expression magnitude; **(C)** heatmap of pathway enrichment analysis in the identified key cell population; **(D–F)** Pseudotime trajectory analysis of the key cell type: each point represents an individual cell coloured by pseudo time (dark blue = early state; light blue = late state). Black circles with numbers denote distinct cell‐state nodes identified during trajectory inference; **(G, H)** dynamic expression changes of BECN1, HIF1A, MFN1, RRAS, and TAX1BP1 along pseudotime in macrophage subtypes, stratified by cell state; **(G)** and macrophage polarization (M1 vs. M2) **(H)**; **(I)** Violin plots showing state-specific expression patterns of the five hub genes.

To determine key PAH-associated cell types, cell cluster proportions were compared between PAH and control samples. Three cell populations - epithelial cells, granulocytes, and monocytes/macrophages were significantly enriched in PAH samples (Figure 6A; Supplementary Table S16). Evaluation of biomarker expression patterns across these clusters (Supplementary Table S16), in conjunction with the communication analyses, indicated that monocytes/macrophages demonstrated the highest signal integration strength, identifying them as the key cell population (Figure 6B).

GSVA comparing M1 and M2 macrophage subtypes indicated distinct functional programs. M1 macrophages presented enrichment in the mitotic spindle, angiogenesis, and DNA repair pathways, whereas M2 macrophages were enriched for the hedgehog signaling, apical surface, and estrogen response late signatures (Figure 6C; Supplementary Table S16).

Pseudotime trajectory reconstruction was performed using Monocle2, based on highly variable genes (q < 0.1), to delineate macrophage differentiation states and lineage bifurcation (Supplementary Table S16). Following branch point 2, cells diverged into State 3 and State 4, both of which were enriched for M2 macrophages, indicating the successful induction of distinct macrophage phenotypes at this branching point (Figures 6D–F). Temporal expression dynamics of the five biomarkers indicated that *BECN1*, *MFN1*, and *RRAS* maintained relatively stable expression across states, whereas *TAX1BP1* expression exhibited a progressive increase over pseudotime, and *HIF1A* expression peaked early before declining (Figures 6G–I).

To further explore the relationship between the identified biomarkers and macrophage subtypes, prior bioinformatics findings (Supplementary Figure S4A) were integrated with single-cell data. Heatmap analysis demonstrated a negative correlation between *RRAS* and M1 macrophages, while *BECN1*, *HIF1A*, *MFN1*, and *TAX1BP1* were positively correlated with M1 macrophages (Supplementary Figure S4B; Supplementary Table S15), this suggests that macrophages may be involved in disease progression in PAH tissues. Because the identified biomarkers were associated with M1 macrophages, we further quantified the M1/M M2 macrophage proportion in PAH tissues (Supplementary Figure S5A) and analyzed the correlations between the five genes (RRAS, BECN1, MFN1, HIF1A and TAX1BP1) and canonical M1/M2 surface markers or functional molecules (Supplementary Figure S5B–D). The data confirmed that all five genes are statistically linked to M1 and/or M2 macrophages.

### 3.6 Validation of mitophagy-related PAH genes using qPCR, WB, and immunofluorescence co-localization

**FIGURE 7:**
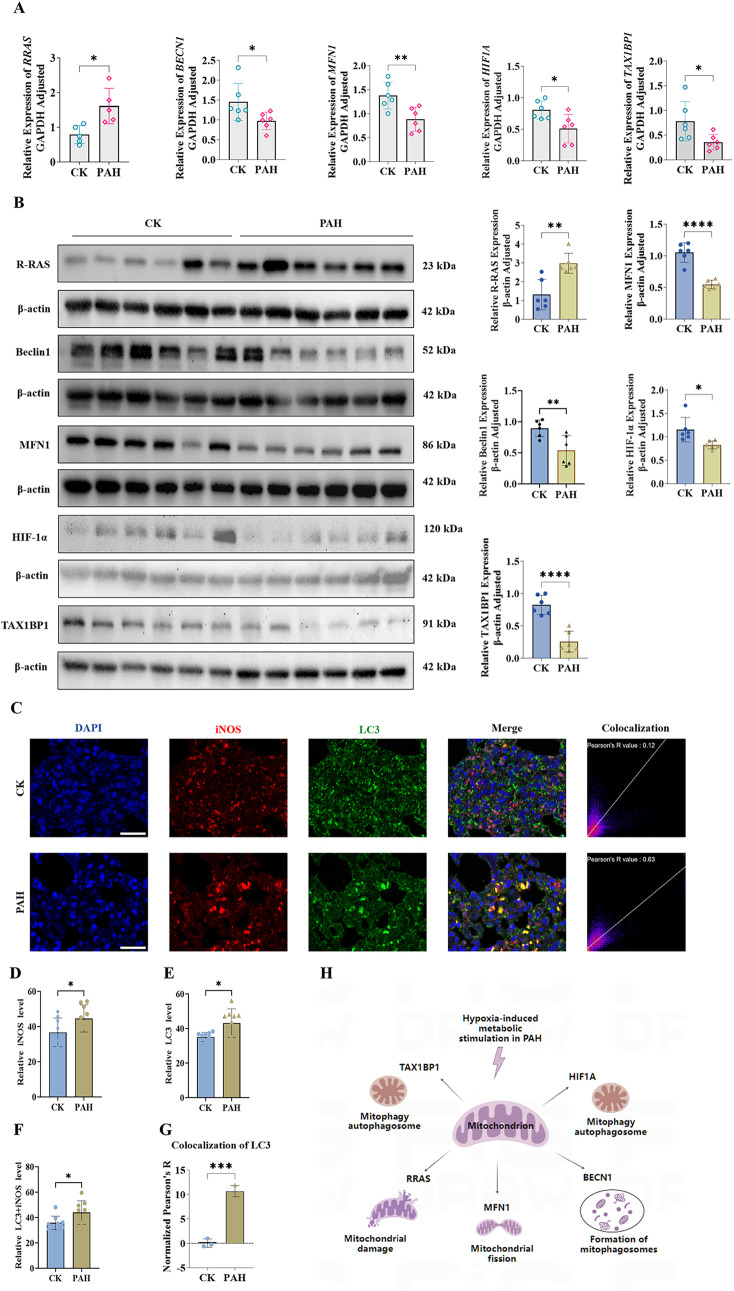
Validation of the Relationship between Biomarkers and Mitochondria. **(A)** expression levels of RRAS, BECN1, MFN1, HIF1A and TAX1BP1 in rat lung tissues detected by qPCR; **(B)** protein expression levels of RRAS, BECN1, MFN1, HIF1A and TAX1BP1 in rat lung tissues detected by WB; **(C)** expression and localization of LC3 and iNOS detected by immunofluorescence double-labeling colocalization analysis; **(D)** relative expression levels of iNOS in lung tissues; **(E)** relative expression levels of autophagy marker LC3 in lung tissues; **(F)** relative colocalization levels of LC3 and iNOS in lung tissues of CK and PAH; **(G)** normalized Pearson’s correlation coefficient (Pearson’s R) for colocalization of LC3 + iNOS in lung tissues Scale bar 100 μm; **(H)** Schematic diagram of the relationship between the five biomarkers and mitochondria. In the figures, ns indicates p > 0.05; *p < 0.05; **p < 0.01; ***p < 0.001; ****p < 0.0001.

To investigate the expression changes of the five biomarkers in PAH, qPCR and WB analyses were conducted on rat lung tissues. Results from both qPCR and WB analyses indicated that, compared with the control (CK) group, the expression level of *RRAS* was significantly upregulated in the PAH group. In contrast, the expression levels of *BECN1*, *HIF1A*, *TAX1BP1*, and *MFN1* were significantly downregulated (Figures 7A,B; Supplementary Table S17; Supplementary Table S18).

Additionally, immunofluorescence double-labeling co-localization analysis was employed to examine the expression and localization of microtubule-associated protein LC3 and iNOS. Compared with the CK, the PAH exhibited significantly increased expression of both iNOS and LC3, accompanied by significantly enhanced co-localization (Pearson’s correlation coefficient R = 0.63). In contrast, minimal co-localization was observed in the CK (Pearson’s correlation coefficient R = 0.12) (Figures 7C–G; Supplementary Table S19). These findings indicate that iNOS may participate in the autophagic pathway and be closely associated with inflammatory responses. Next, combining the qPCR results with the immunofluorescence detection of the M1 marker iNOS, we assessed the correlation between each candidate gene and iNOS. Pearson correlation analysis (p < 0.05) demonstrated a positive correlation between RRAS and iNOS, while BECN1, MFN1, HIF1A and TAX1BP1 were negatively correlated with iNOS (Supplementary Figure S5E; Supplementary Table S20). These findings indicate that the identified genes may differentially regulate M1/M2 polarization and macrophage functional status in PAH.

From these results, a schematic diagram was constructed to depict the relationship between the five biomarkers and mitochondrial processes in PAH (Figure 7H). Collectively, the identified mitochondrial-related genes were expressed in macrophages, indicating that PAH may influence their expression, thereby affecting mitochondrial processes such as mitophagy, mitochondrial fission, and autophagosome formation. These findings indicate that the five biomarkers play critical roles in the pathogenesis of PAH, potentially through the dysregulation of mitophagy and its impact on disease progression.

## 4 Discussion

In this study, the relationship between PAH and mitophagy was systematically investigated through the integration of bioinformatics analysis, animal experiments, and scRNA-seq. Five key biomarkers involved in the pathogenesis of PAH (RRAS, BECN1, MFN1, HIF1A, TAX1BP1) were ultimately identified, providing new evidence for understanding the molecular mechanisms of PAH and developing diagnostic/therapeutic strategies. The discussion covers mitophagy in PAH, candidate gene screening and validation, biomarker function, immune microenvironment interplay, single-cell macrophage insights, and mechanistic experiments.

From the analysis of the GSE113439 dataset, a total of 2,753 DEGs associated with PAH were identified. KEGG pathway enrichment analysis indicated that these genes were closely linked to the mitophagy pathway. This result is consistent with previous studies reporting the key involvement of mitochondrial dysfunction in the initiation and progression of PAH (Zhang et al., 2025; Zhang et al., 2022; Colon Hidalgo et al., 2022). Mitophagy has attracted increasing attention since its initial characterization (Lemasters, 2005). Physiological mitophagy preserves mitochondrial homeostasis; in PAH its dysregulation amplifies oxidative stress, drives vascular remodeling and hastens disease progression (D'Arcy, 2024). MCT-induced PAH rats confirmed mitophagy activation as a maladaptive stress response, yet its stage-specific regulation remains unclear (Mao et al., 2023; Chen et al., 2018).

WGCNA of PAH lungs intersected with DEGs and MRGs yielded 13 candidates enriched for GDP/GTP binding, GTPase activity, mitophagy and neurodegeneration pathways. WGCNA of PAH lung tissue intersected with DEGs and MRGs yielded 13 candidates enriched for GDP/GTP binding, GTPase activity, mitophagy and neurodegeneration pathways. Consistent with previous reports (such as BECN1 being a key regulator of autophagy (Liang et al., 1999; Fernández et al., 2018), HIF1A being involved in hypoxia induced vascular remodeling (Dai et al., 2024), and MFN1 maintaining mitochondrial dynamics and homeostasis) (Tábara et al., 2025).

PPI network analysis pinpointed RRAS, BECN1, MFN1, HIF1A and TAX1BP1 as central hubs linking mitophagy to PAH. Applying five machine-learning algorithms (SVM, GLM, NN, RF, XGBoost) to the training cohort and validating in GSE53408 identified these five genes as robust diagnostic biomarkers with AUC >0.90. The relevant biomarkers identified in this study reveal the pathological mechanisms of pulmonary vascular remodeling, mitochondrial dysfunction, and macrophage activation in PAH, and exploring their expression characteristics in peripheral blood mononuclear cells (PBMCs) can provide a supplement for the development of non-invasive detection methods. Recent studies on high-altitude PAH (HAPH, an important subtype of PAH) have shown (Wu et al., 2023). This study showed that C1 (atypical) and C2 (intermediate) monocyte subsets were enriched in PBMCs of HAPH patients, and the expression of HIF-1 α was significantly reduced. This is consistent with the downregulation trend of HIF-1A in PAH rat lung tissue in this study, suggesting that abnormal expression of HIF-1A may exhibit a similar pattern in PBMCs. In addition, the study suggests that HAPH has a common immune adaptation mechanism with other types of PH, which also supports the possibility of the markers identified in this study maintaining consistent expression in PBMCs. I plan to include PBMC samples from PAH patients in the future to clarify the tissue peripheral blood expression association of these biomarkers and further extend the clinical value of the biomarkers in this study.

Among the identified biomarkers, *RRAS*, a member of the RAS superfamily and a well-established oncogene, represented a novel finding in the context of PAH, particularly in relation to mitophagy (Liu et al., 2017; Weber and Carroll, 2021). Traditionally, *RRAS* has been associated with cell proliferation and tumorigenesis via modulation of the MAPK and PI3K/AKT signaling pathways (Bahar et al., 2023). However, through WGCNA, machine learning, and validation using quantitative PCR and WB, *RRAS* was significantly upregulated in lung tissues from patients with PAH, indicating a potential non-oncogenic pathological role in the disease. Although direct evidence linking *RRAS* to PAH pathogenesis remains limited, its identification as a mitophagy-related biomarker in osteoarthritis supports its possible involvement in mitochondrial quality control beyond its classical role in cancer biology (Ruan et al., 2024).

Single-cell data linked RRAS to M1-macrophage state transitions; dual immunofluorescence showed RRAS upregulation coinciding with enhanced iNOS/LC3 co-localization (PAH R = 0.63 vs. control R = 0.12), implying RRAS-driven mitophagy modulation via inflammation. Concordant CIBERSORT analyses revealed expanded M1 infiltrates in PAH, and published evidence indicates RRAS can trigger NF-κB–dependent IL-6/TNF-α release to promote vascular remodeling and elevated pulmonary pressures (Tago et al., 2019).

Further correlation analysis between biomarker expression and immune cell populations demonstrated a positive association between *RRAS* and activated dendritic cells (DCs) as well as eosinophils. DCs, which serve as central regulators of immune responses, have been increasingly implicated in PAH pathogenesis (van Uden et al., 2021; Koudstaal et al., 2020).

It can induce T cell differentiation into Th17 cells, and the imbalance of Th17/regulatory T cells is involved in the occurrence of hypoxia induced, chronic obstructive pulmonary disease related, and connective tissue disease-related PAHs (Zhu et al., 2019). In addition, recent studies have found that the peripheral blood eosinophil count of PAH patients is reduced, which may play a protective role by releasing lipid mediators such as 14-HDHA and 17-HDHA to promote regression, reduce inflammatory cell infiltration, and maintain the homeostasis of pulmonary artery smooth muscle cells (Shu et al., 2023).

These observations provide novel insights into the potential role of *RRAS* in cardiovascular disease and highlight the need for further mechanistic studies.

GSEA indicated that *RRAS*, *BECN1*, *MFN1*, *HIF1A*, and *TAX1BP1* were significantly enriched in the spliceosome and neuroactive ligand–receptor interaction pathways. Dysregulation of spliceosome function has been implicated in cardiovascular disease and is also recognized as a contributor to mitochondrial dysfunction (Cao et al., 2024; Zhou et al., 2025). The neuroactive ligand–receptor interaction pathway may participate in the neuroendocrine dysregulation observed in PAH (Chinnappan et al., 2019).

ScRNA-seq of GSE210248 identified monocytes/macrophages as the dominant PAH-associated population; GSVA showed M1 activation via mitotic-spindle/angiogenesis pathways and M2 differentiation via hedgehog signaling, underscoring subset heterogeneity. Pseudotime trajectories revealed stable BECN1/MFN1/RRAS, rising TAX1BP1, and biphasic HIF1A, with RRAS negatively and the other four genes positively correlated with M1 signatures, implying distinct regulatory roles. qPCR/WB validated RRAS upregulation and downregulation of BECN1, MFN1, HIF1A and TAX1BP1 in PAH lungs, while immunofluorescence showed enhanced iNOS/LC3 co-localization (R = 0.63), indicating inflammatory-autophagy crosstalk (de Lavera et al., 2017). Collectively, these findings validate the functional significance of the identified biomarkers and indicate that mitophagy dysfunction, potentially driven by inflammatory dysregulation, may contribute to the pathological progression of PAH (Marchi et al., 2023).

This study is based on lung tissue validation. Although it can accurately reflect the core pathological features of mitochondrial dysfunction and macrophage activation, lung biopsy is not a routine examination for PAH patients and poses a key challenge to biomarker transformation. Previous studies have confirmed that bronchoalveolar lavage fluid (BALF) can be used as a minimally invasive sample for lung marker detection in acute respiratory distress syndrome (ARDS) subtypes (Sathe et al., 2023), and can also detect pulmonary macrophage derived markers (such as Fizz1) associated with vascular remodeling in hypoxia induced PAH (HPH, PAH subtypes) (Li et al., 2024). Subsequently, lung monocytes/macrophages enriched in BALF can be utilized to establish their expression association with lung tissue markers through ultra sensitive techniques, and sample processing can be optimized by combining PAH pathology. If it can be confirmed that the levels of biomarkers in BALF are correlated with clinical indicators of PAH, they can be converted into minimally invasive detection indicators, which can avoid the invasiveness of lung biopsy while retaining accurate reflection of the pathological status of PAH, laying the foundation for clinical application.

Although our single-cell analysis was limited to lung tissue, it remains unclear whether the five mitophagy-related biomarkers exhibit similar M1/M2-associated expression patterns in peripheral blood mononuclear cells (PBMCs) of PAH patients. This tissue specificity question is critical for translating our findings into minimally invasive diagnostic tools. Future studies should therefore compare the transcriptional profiles of circulating monocytes/macrophages with their pulmonary counterparts to determine if PBMCs can serve as a surrogate for lung-based biomarker assessment.

## 5 Conclusion

Integrative analyses revealed a strong association between PAH and mitophagy, identifying *RRAS*, *BECN1*, *MFN1*, *HIF1A*, and *TAX1BP1* as potential diagnostic biomarkers. These findings contribute to the theoretical understanding of the pathological mechanisms involved in PAH and offer novel perspectives for the development of diagnostic and therapeutic strategies. Further research should focus on clinical validation and targeted intervention studies to facilitate the translational application of these biomarkers in clinical practice.

## 6 Limitations and future directions

Several limitations of this study should be acknowledged. The heterogeneity of PAH may affect biomarker expression patterns, underscoring the need for more refined, stratified analyses in future research. Additionally, the underlying molecular mechanisms require further validation by genetic knockout models or pharmacological interventions. Future research should prioritize clinical validation and interventional studies to support the translational application of these biomarkers in the clinical management of PAH.

## Data Availability

The data presented in this study are openly available in the Gene Expression Omnibus (GEO) repository (https://www.ncbi.nlm.nih.gov/geo/) under the accession numbers GSE210248, GSE113439, GSE130391, and GSE53408. The list of mitophagy-related genes (MRGs) analyzed in this study was retrieved from the MitoCarta 3.0 database (https://www.broadinstitute.org/mitocarta).

## Supplementary material

SUPPLEMENTARY FIGURE S1 WGCNA analysis. **(A)** WGCNA analysis showing no abnormal samples; **(B)** WGCNA analysis identifying 14 expression modules; **(C)** Correlation diagram between different immune cells; **(D)** Correlation heatmap of PAH in different immune cells; **(E)** Validation of biomarker expression in the validation datasets; **(F)** ROC curves for each biomarker in the validation set. Data are presented as mean ± SEM. ns, p > 0.05; *p < 0.05; **p < 0.01; ***p < 0.001; ****p < 0.0001.

SUPPLEMENTARY FIGURE S2 Single-cell sequencing analysis. **(A,B)**: Quality control and preprocessing of single-cell RNA sequencing data; **(C)** Volcano plot of highly variable gene screening; **(D)** Fragmentation plot of principal component analysis for single-cell samples; **(E)**: Line plot of principal component analysis; **(F)** Two-dimensional scatter plot of principal component analysis (PCA); **(G)** Gene enrichment heatmap.

SUPPLEMENTARY FIGURE S3 Cell–cell communication network analysis among vascular and immune cell populations. **(A, B)** Global intercellular communication network of the analyzed cell populations. Each node represents a specific cell type, and the edge thickness indicates the interaction strength between sender and receiver cells. The color and size of the nodes reflect the interaction strength and total communication probability, respectively; **(C, D)** Quantitative analysis of outgoing and incoming interaction strength of each cell type. The x-axis represents outgoing interaction strength (signal sending capacity), and the y-axis represents incoming interaction strength (signal receiving capacity). Node size indicates the total number of interactions; **(E)** Representative ligand–receptor signaling networks, including CCL, CXCL, SPP1, and MIF pathways, demonstrating the major communication axes among fibroblasts, macrophages, smooth muscle cells, and T cells. Thicker lines represent stronger communication probabilities between cell types; **(F)** Heatmap showing the roles of different cell types in the CCL signaling pathway, categorized as sender, receiver, mediator, or influencer. The intensity of the color corresponds to the interaction strength.

SUPPLEMENTARY FIGURE S4 Flowchart of biomarker screening and correlation with key cells. **(A)** Flowchart for screening BECN1, HIF1A, MFN1, RRAS, and TAX1BP1; **(B)** Correlation analysis of BECN1, HIF1A, MFN1, RRAS, and TAX1BP1 with M1-type macrophages. The colour and size of the circles indicate the correlation of gene expression, where the colour ranges from blue to red, representing the correlation from negative to positive, and the larger the circle, the stronger the correlation.

SUPPLEMENTARY FIGURE S5 Correlation between M1/M2 macrophages and PAH, as well as five candidate genes. **(A)** Bar chart of the M1/M2 ratio of macrophages in PAH and control tissues calculated based on single-cell bioinformatics analysis; **(B)** Bubble plot of the expression of 5 candidate genes (RRAS, BECN1, MFN1, HIF1A, TAX1BP1) in M1/M2 macrophages; **(C)** Correlation heatmap between 5 candidate genes (RRAS, BECN1, MFN1, HIF1A, TAX1BP1) and typical markers of M1/M2 (such as M1: CCL5, M2: CD163, MRC1) (correlation coefficient r is represented by color, −0.5 is significant negative correlation, 1.0 is significant positive correlation); **(D)** The correlation heatmap between 5 candidate genes (RRAS, BECN1, MFN1, HIF1A, TAX1BP1) and functional factors (such as M1: IDO1, MMP9, IRF1, IRF5, CXCL9, M2: TGFβ family, CCL22, LYVE1, VEGF family, etc.) (correlation coefficient r is represented by color, −0.5 is a significant negative correlation, 1.0 is a significant positive correlation) ^∗^p < 0.05; ^∗∗^p < 0.01; ^∗∗∗^p < 0.001; **(E)** Scatter plots (correlation coefficient R2) of 5 candidate genes (RRAS, BECN1, MFN1, HIF1A, TAX1BP1) and iNOS.

## Abbreviations

AUC, Area Under the Curve; DEGs, Differentially expressed genes; GLM, Generalized linear model; GO, Gene Ontology; GSVA, Gene set variation analysis; HE, Hematoxylin and Eosin; HIF1A, Hypoxia-Inducible Factor 1 Subunit Alpha; KEGG, Kyoto Encyclopedia of Genes and Genomes; LC3, Microtubule-Associated Protein 1 Light Chain 3; MCT, monocrotaline; MFN1, Mitofusin-1; MRGs, Mitophagy-related genes; NN, Neural Network; PAAT, Pulmonary Artery Acceleration Time; PAET, Pulmonary Artery Ejection Time; PAH, Pulmonary Arterial Hypertension; PPI, Protein-protein interaction; qPCR, Quantitative Polymerase Chain Reaction; RF, Random Forest; ROC, Receiver Operating Characteristic; RRAS, RAS Related; scRNA-seq, single-cell RNA sequencing; SD, Sprague-Dawley; SVM, Support Vector Machine; TAX1BP1, Tax1-Binding Protein 1; TGF-β, Transforming growth factor-beta; WB, Western Blot; WGCNA, Weighted gene co-expression network analysis; XGBoost, eXtreme Gradient Boosting.
