# Supplementary figures and images for: Mitophagy-associated biomarkers and macrophage involvement in pulmonary arterial hypertension: identification and functional implications
Source: Front Physiol. 2025 Nov 5;16:1673181. doi: 10.3389/fphys.2025.1673181 (PMC12627872; doi:10.3389/fphys.2025.1673181)

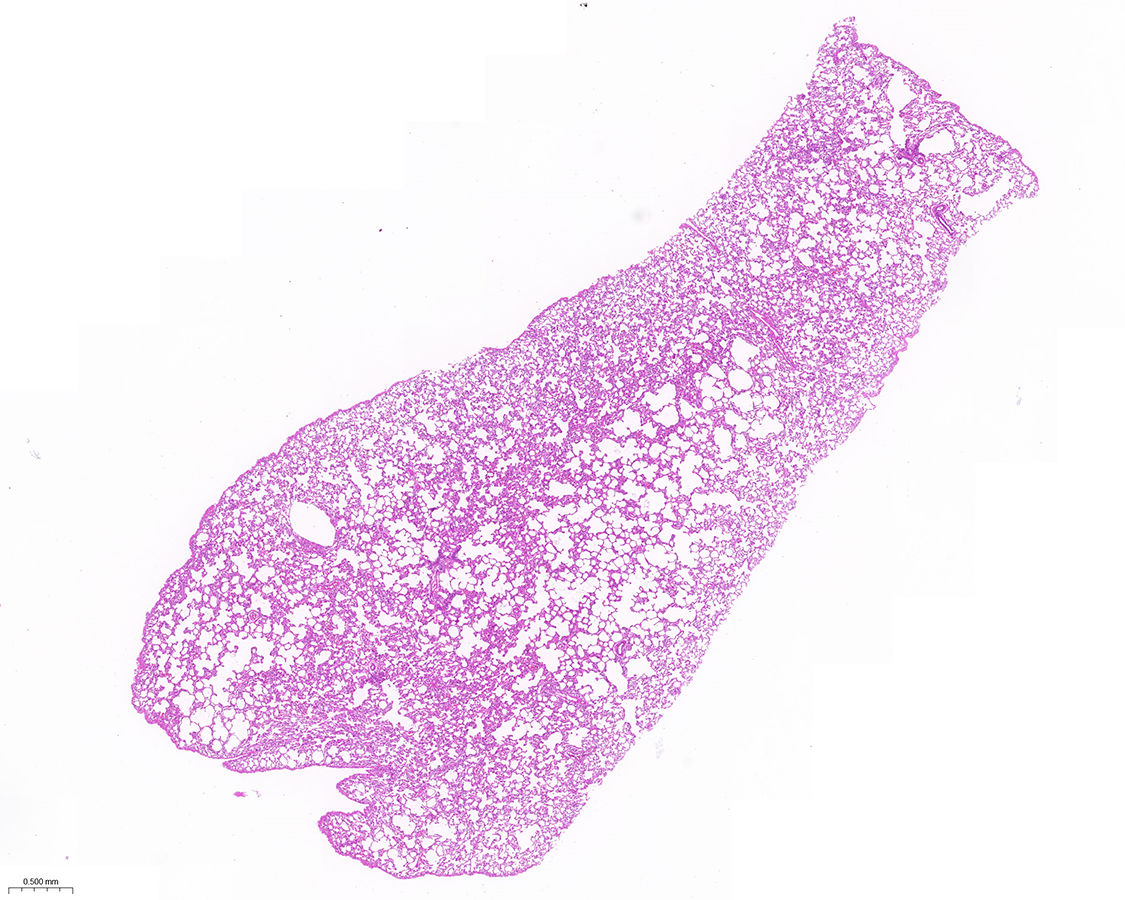

Supplement: Supplementary file 3 [file DataSheet3.zip › Original image of microscope-HE/CK 1.tif]

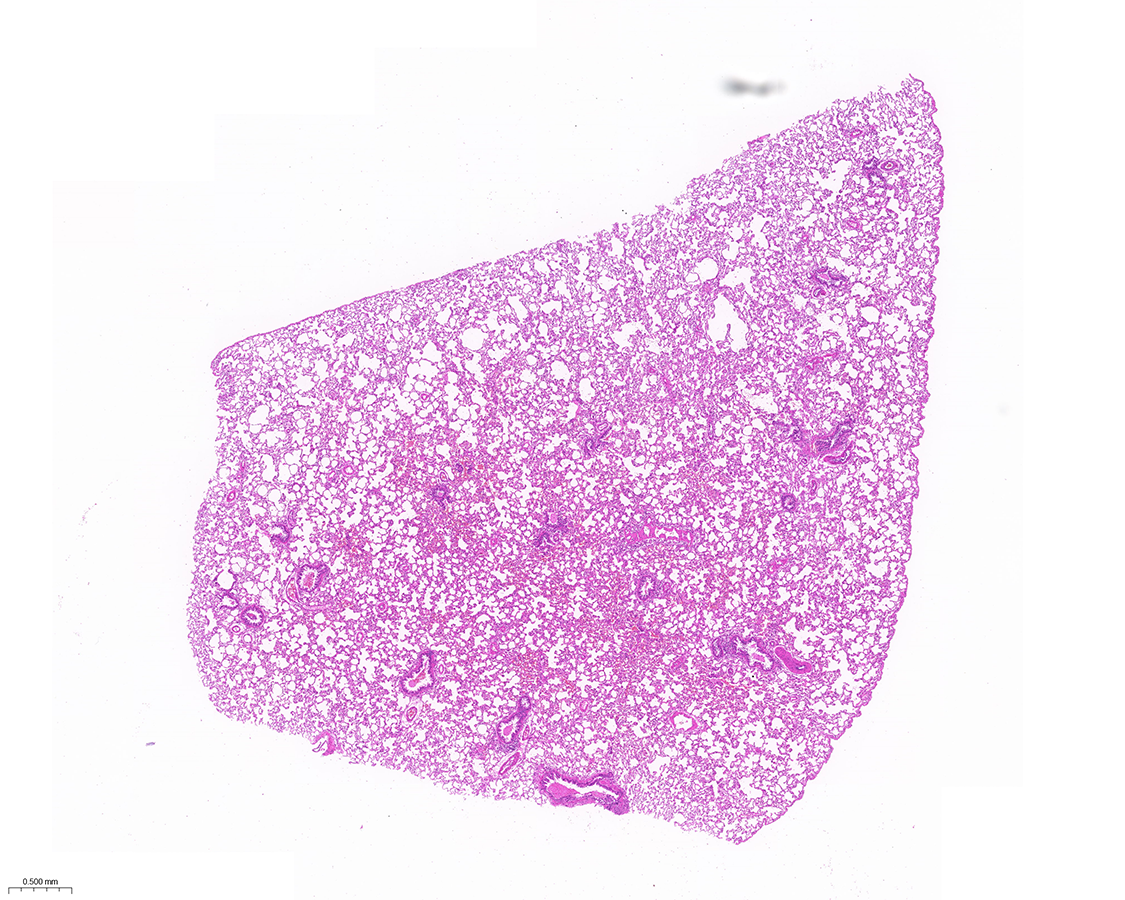

Supplement: Supplementary file 3 [file DataSheet3.zip › Original image of microscope-HE/CK 2.tif]

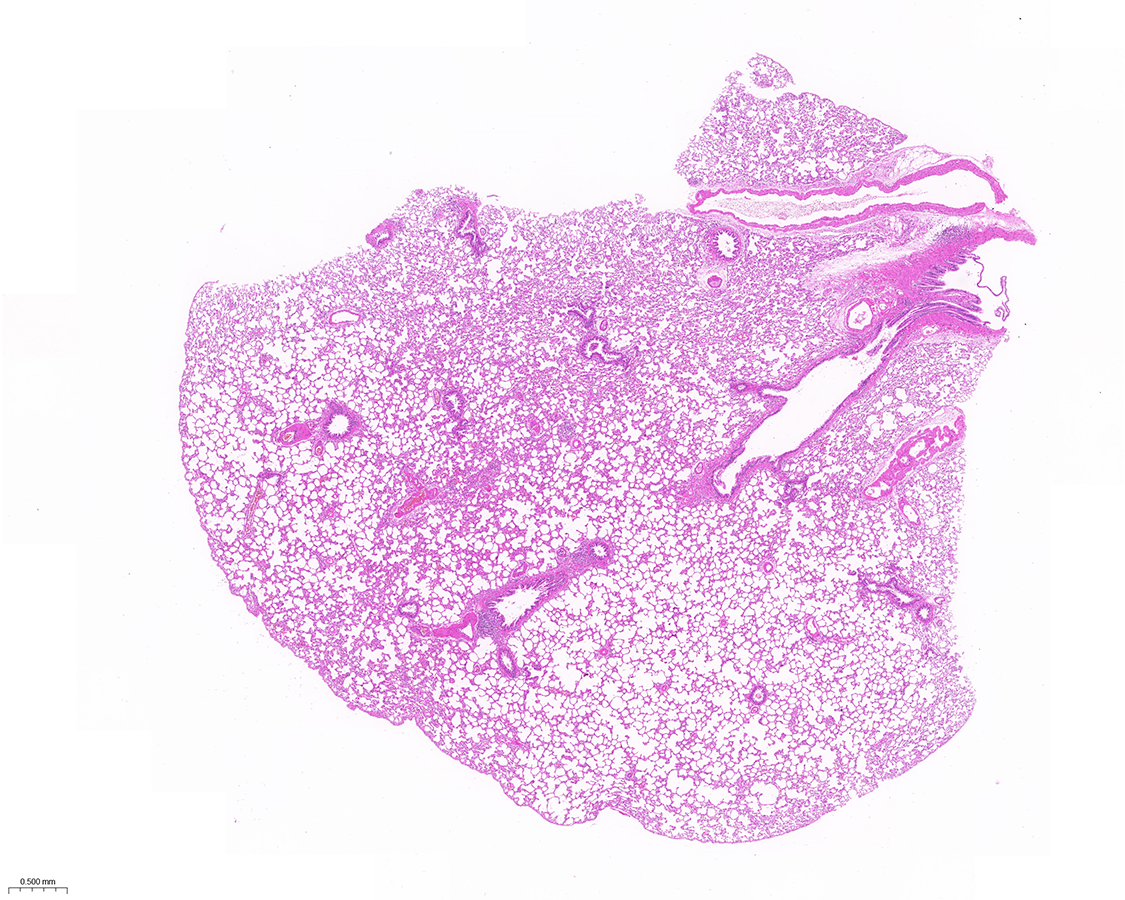

Supplement: Supplementary file 3 [file DataSheet3.zip › Original image of microscope-HE/CK 3.tif]

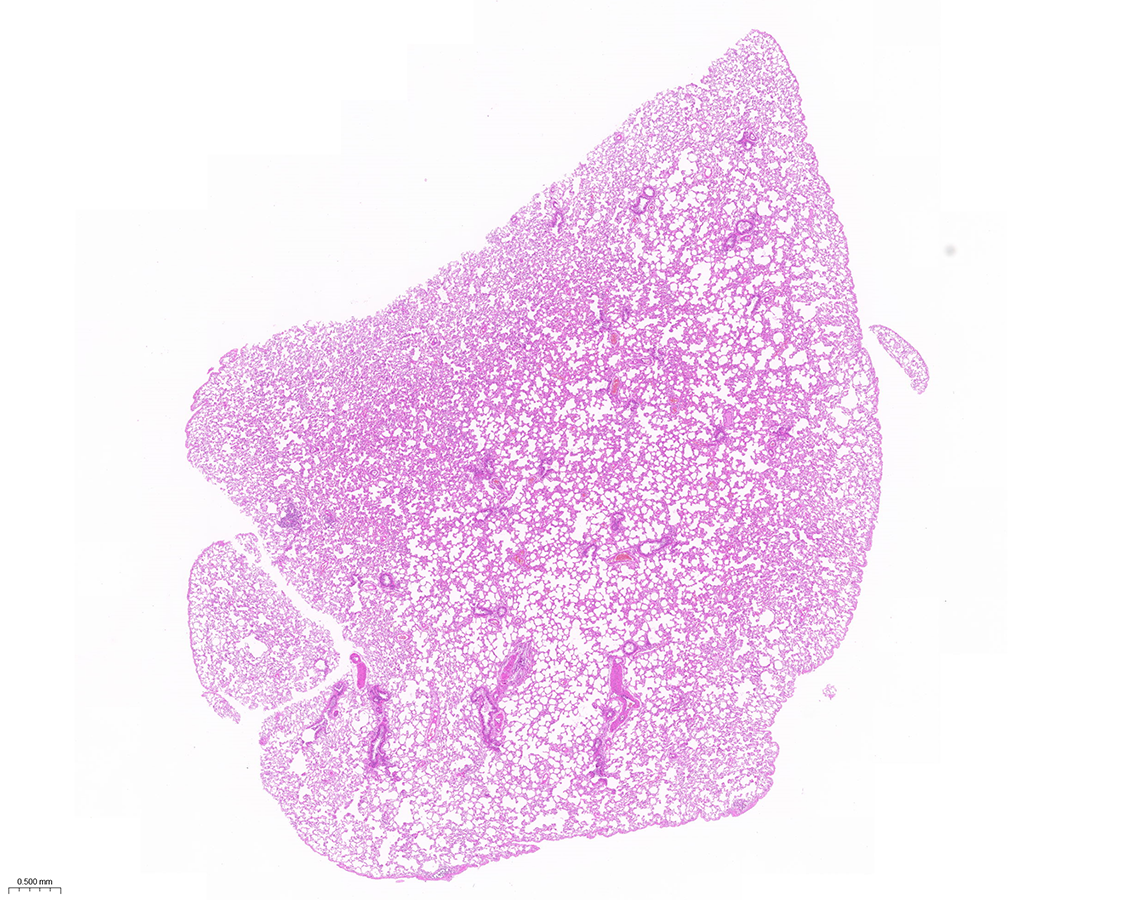

Supplement: Supplementary file 3 [file DataSheet3.zip › Original image of microscope-HE/CK 4.tif]

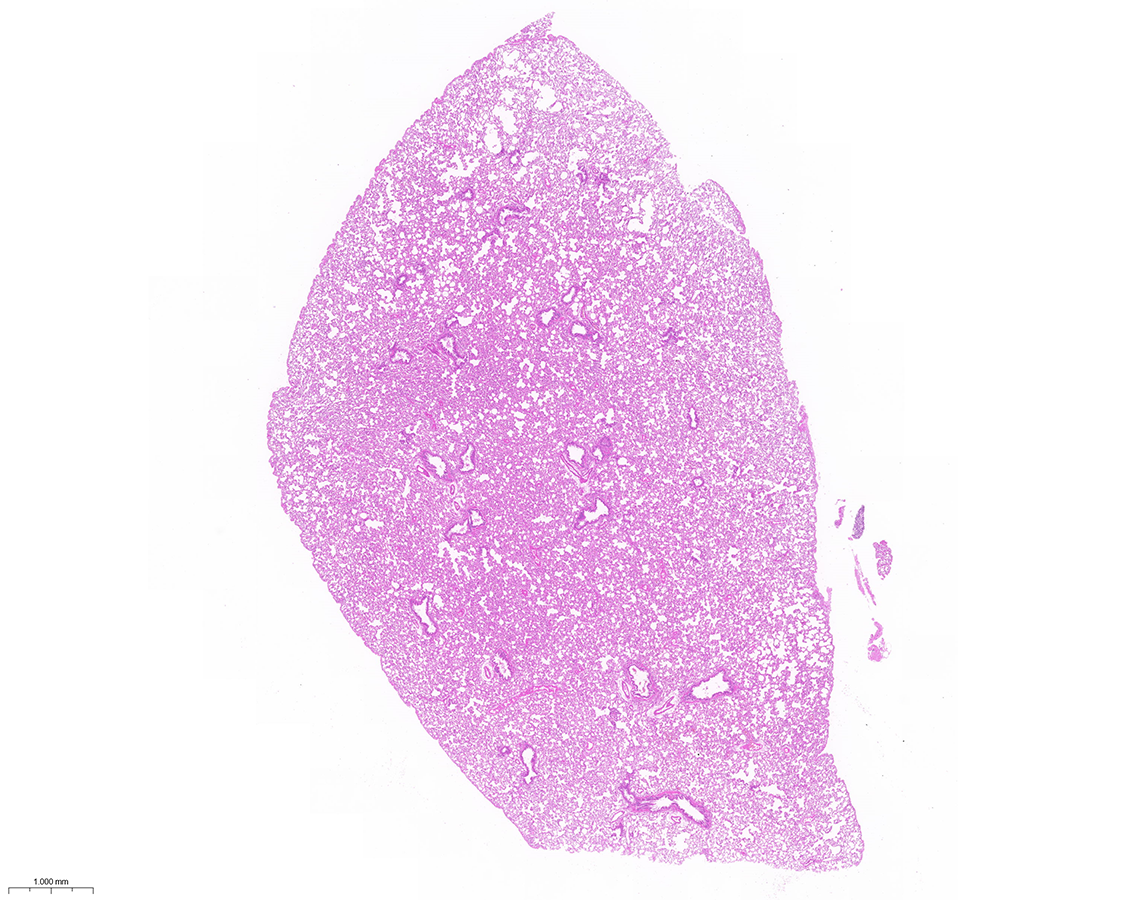

Supplement: Supplementary file 3 [file DataSheet3.zip › Original image of microscope-HE/CK 5.tif]

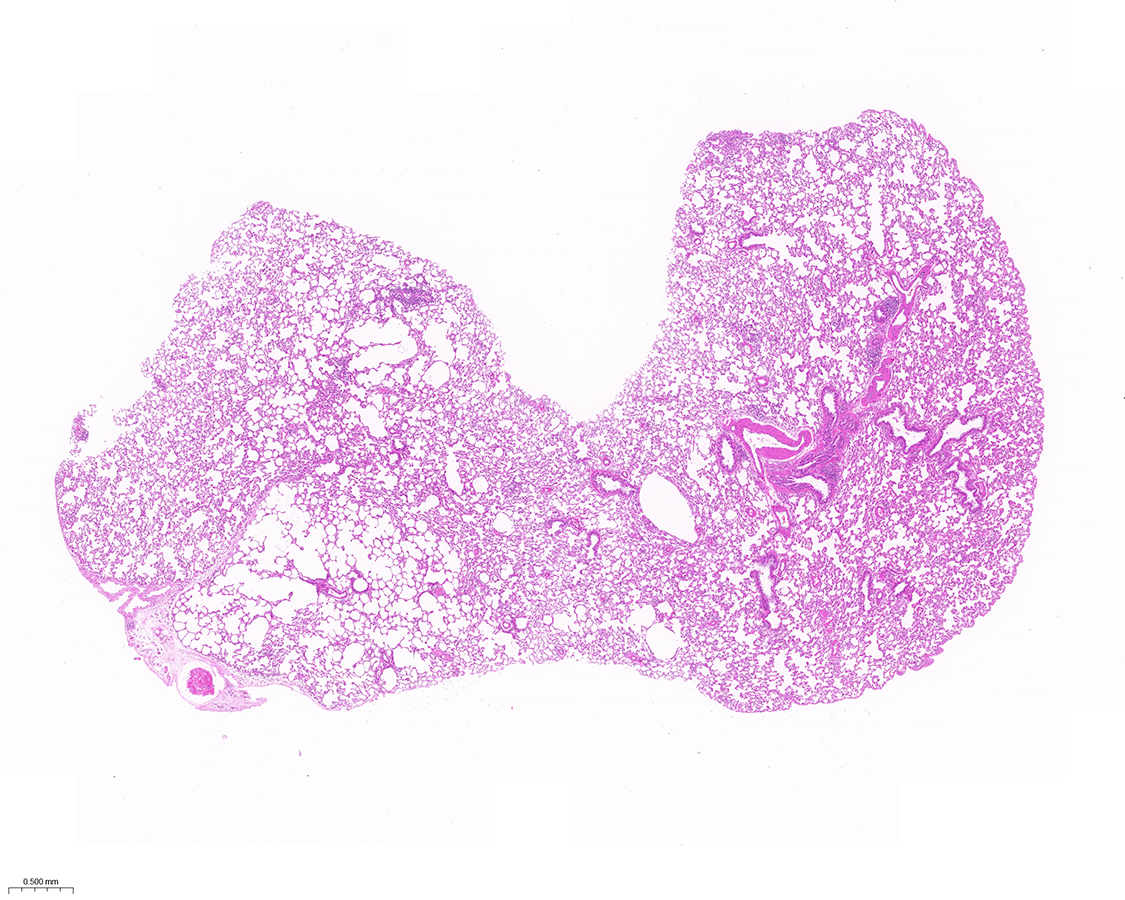

Supplement: Supplementary file 3 [file DataSheet3.zip › Original image of microscope-HE/CK 6.tif]

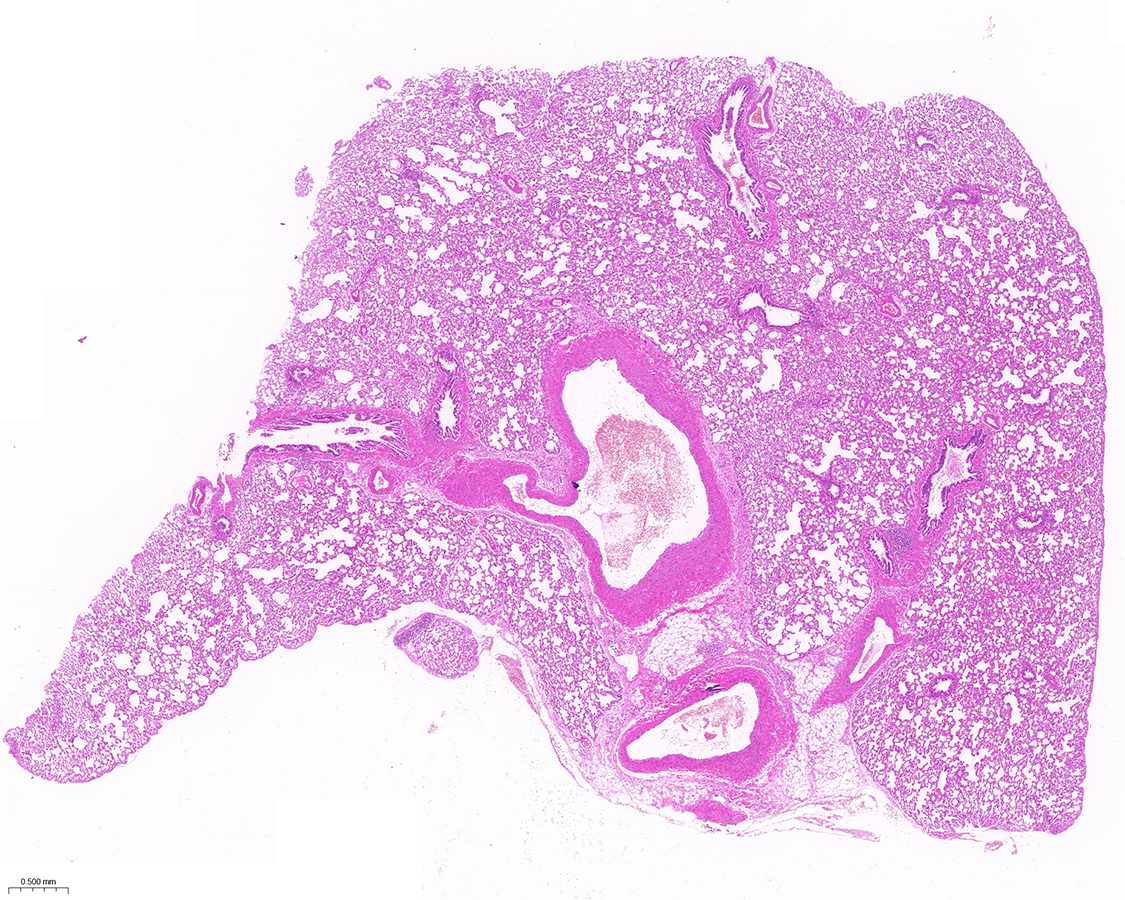

Supplement: Supplementary file 3 [file DataSheet3.zip › Original image of microscope-HE/PAH 1.tif]

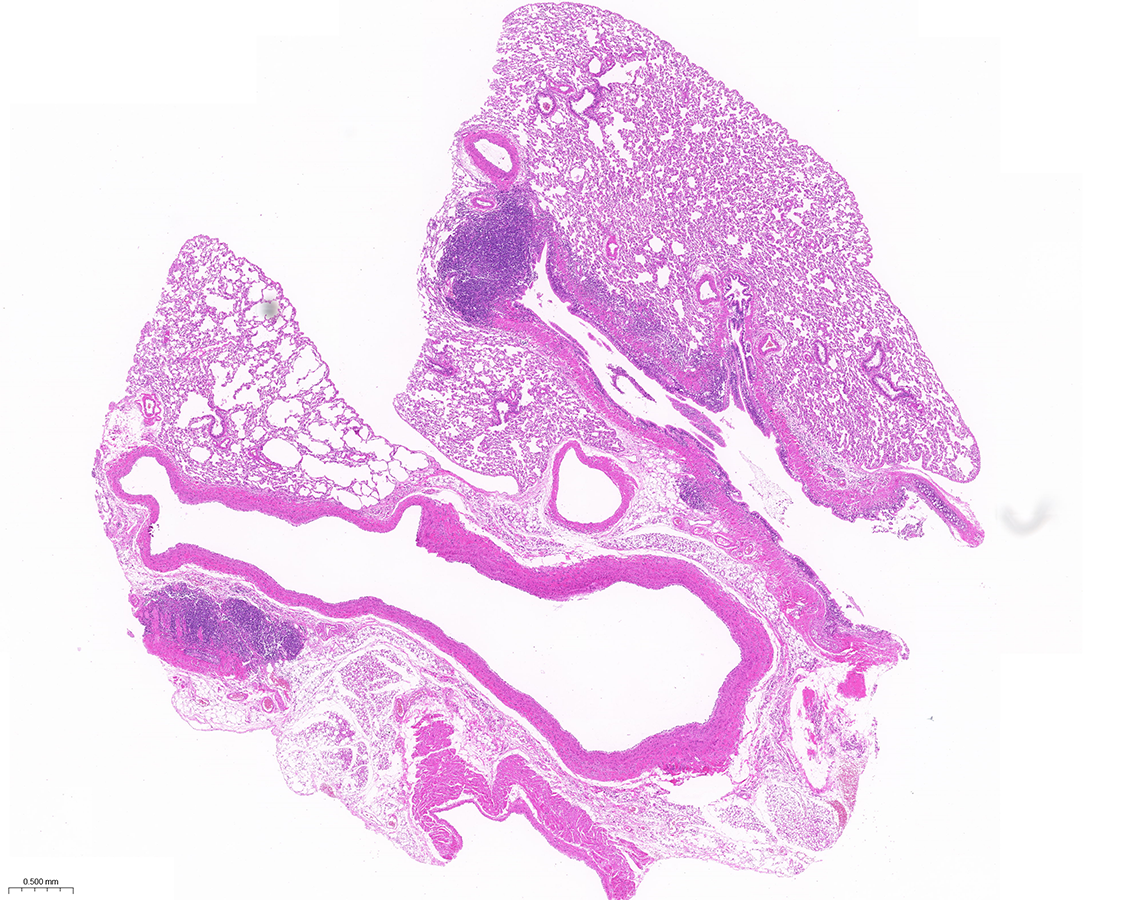

Supplement: Supplementary file 3 [file DataSheet3.zip › Original image of microscope-HE/PAH 2.tif]

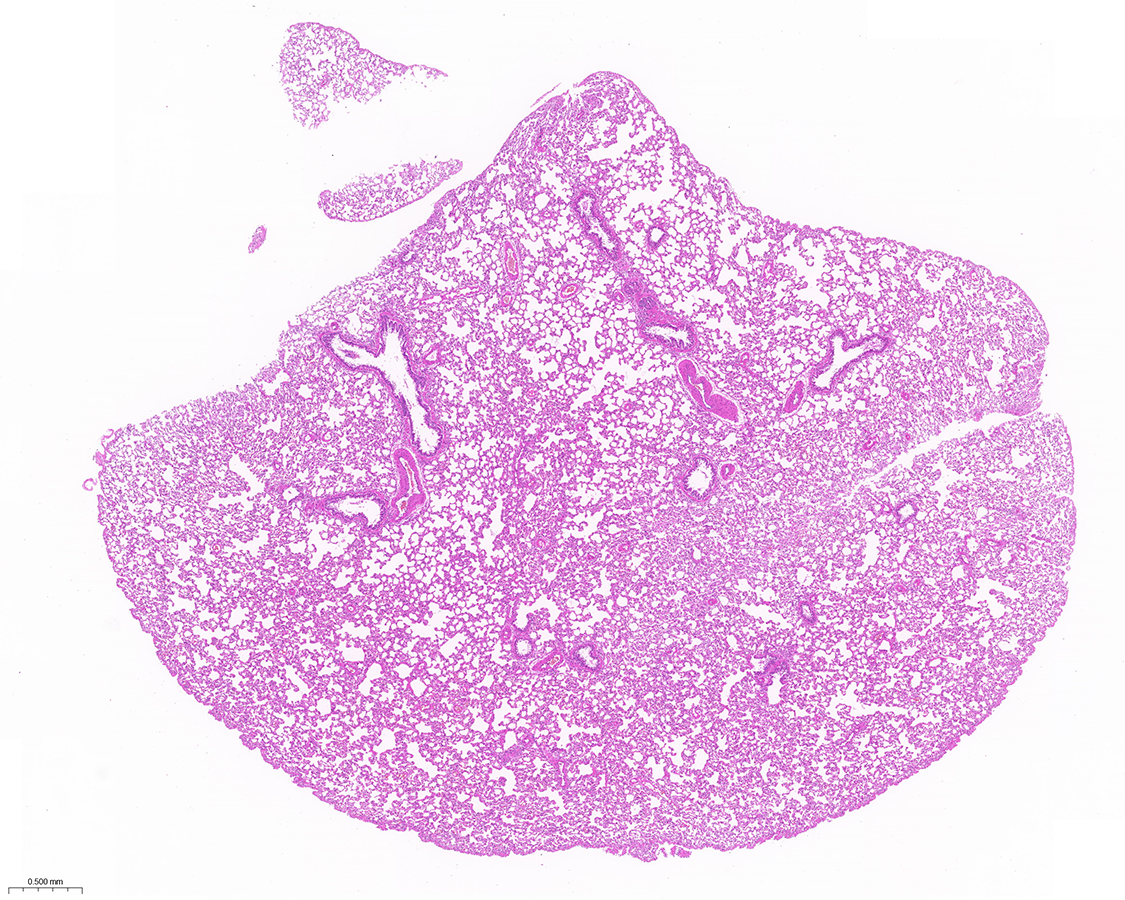

Supplement: Supplementary file 3 [file DataSheet3.zip › Original image of microscope-HE/PAH 3.tif]

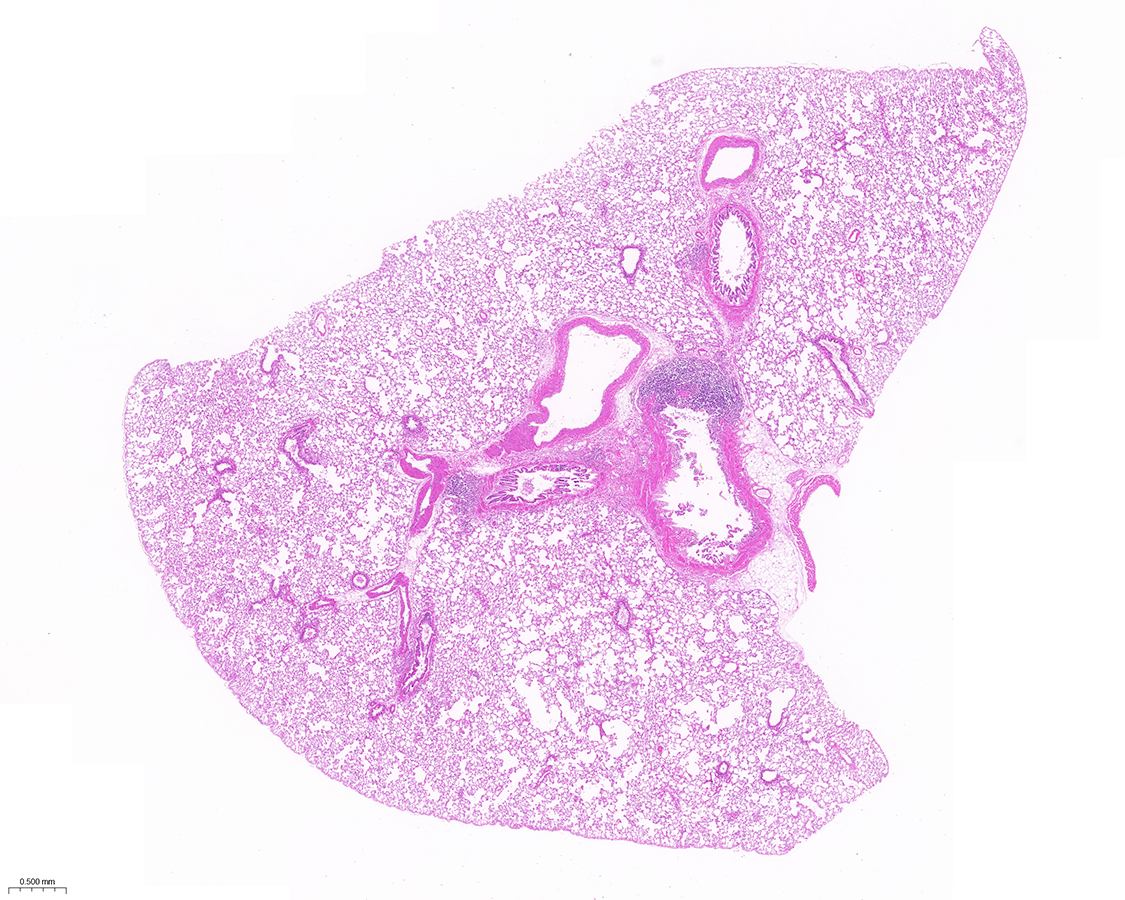

Supplement: Supplementary file 3 [file DataSheet3.zip › Original image of microscope-HE/PAH 4.tif]

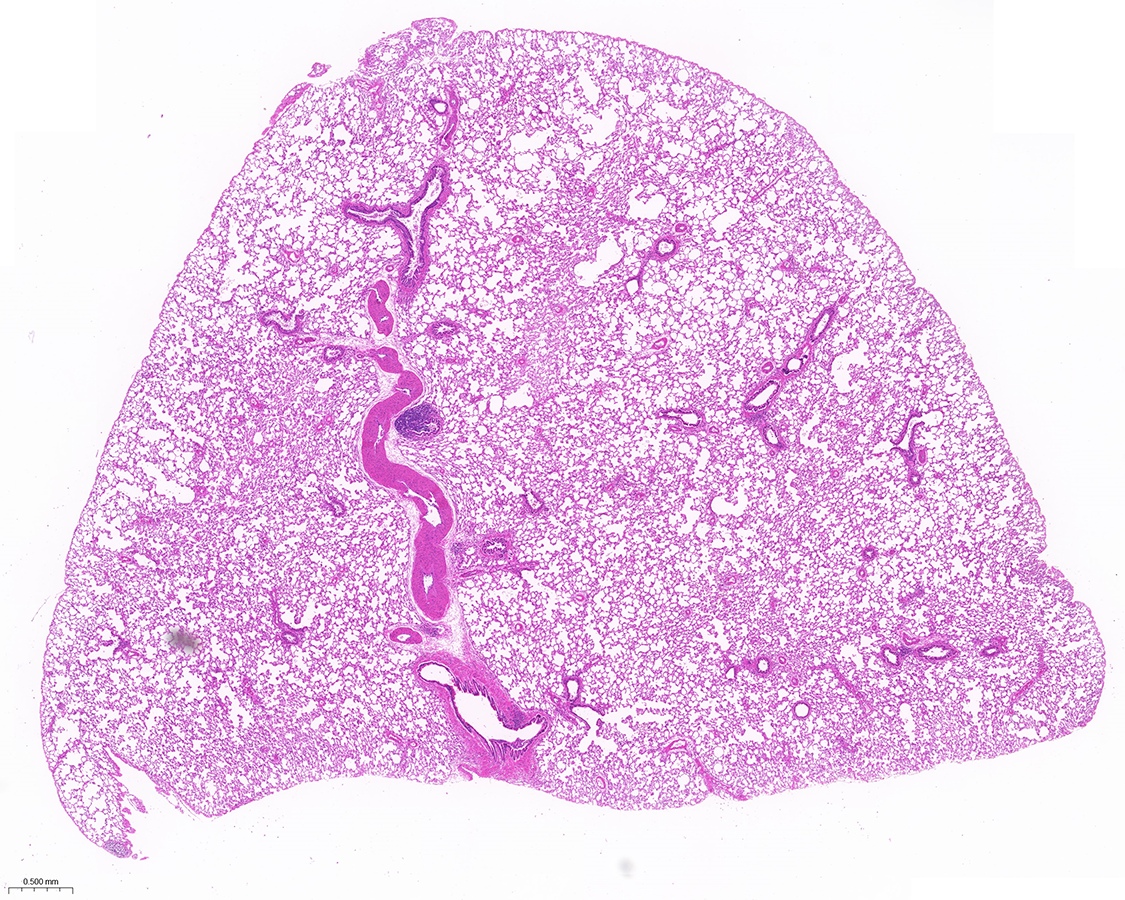

Supplement: Supplementary file 3 [file DataSheet3.zip › Original image of microscope-HE/PAH 5.tif]

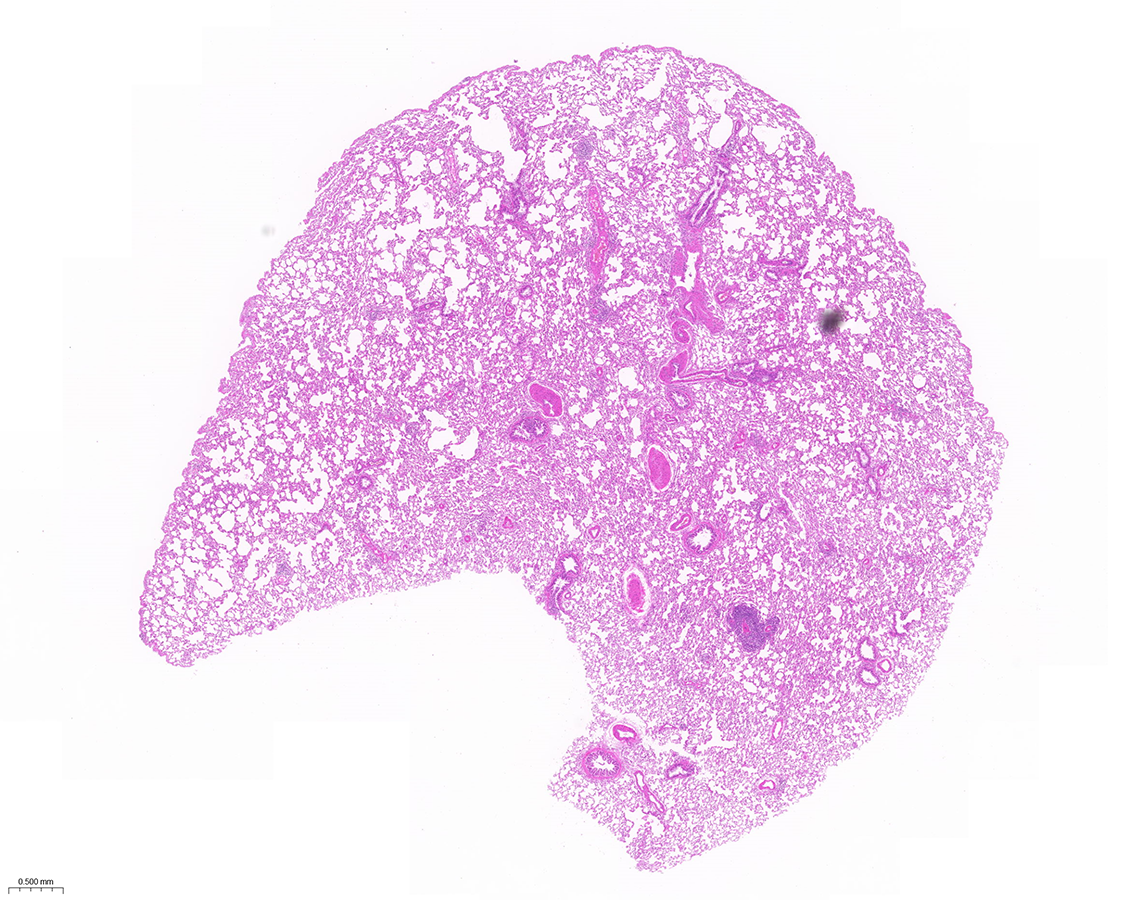

Supplement: Supplementary file 3 [file DataSheet3.zip › Original image of microscope-HE/PAH 6.tif]

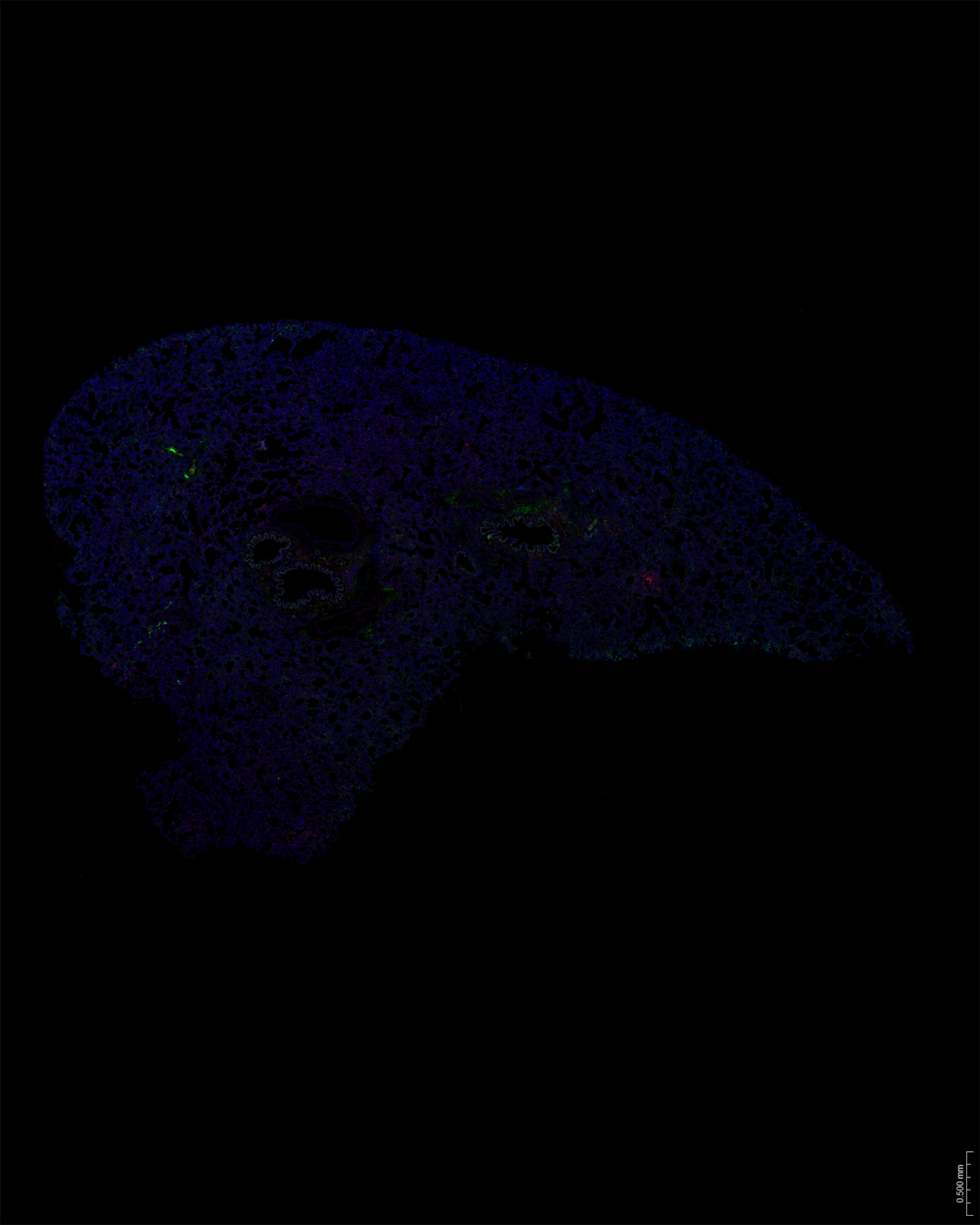

Supplement: Supplementary file 7 [file DataSheet4.zip › Original image of microscope-IF/CK 1.tif]

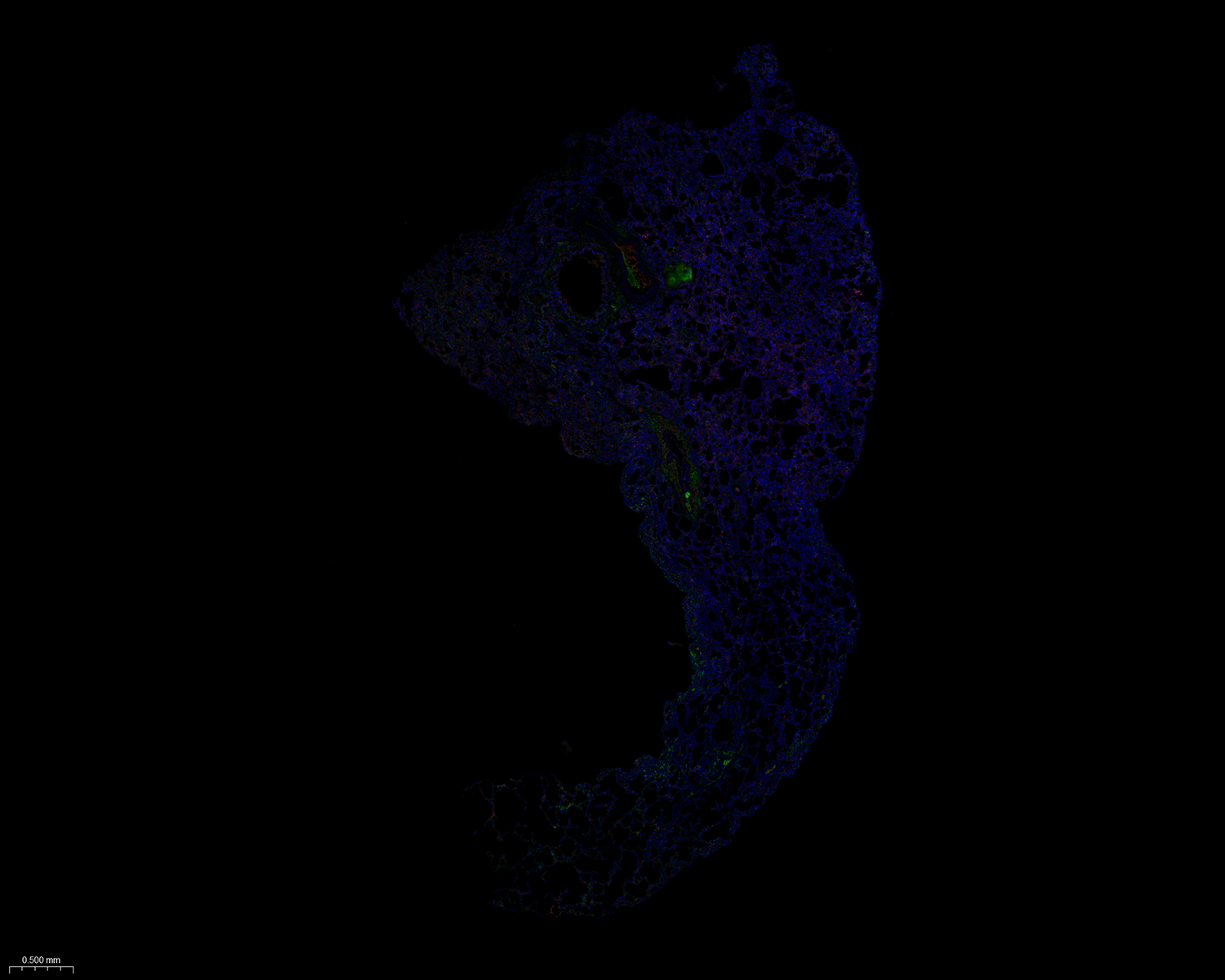

Supplement: Supplementary file 7 [file DataSheet4.zip › Original image of microscope-IF/CK 2.tif]

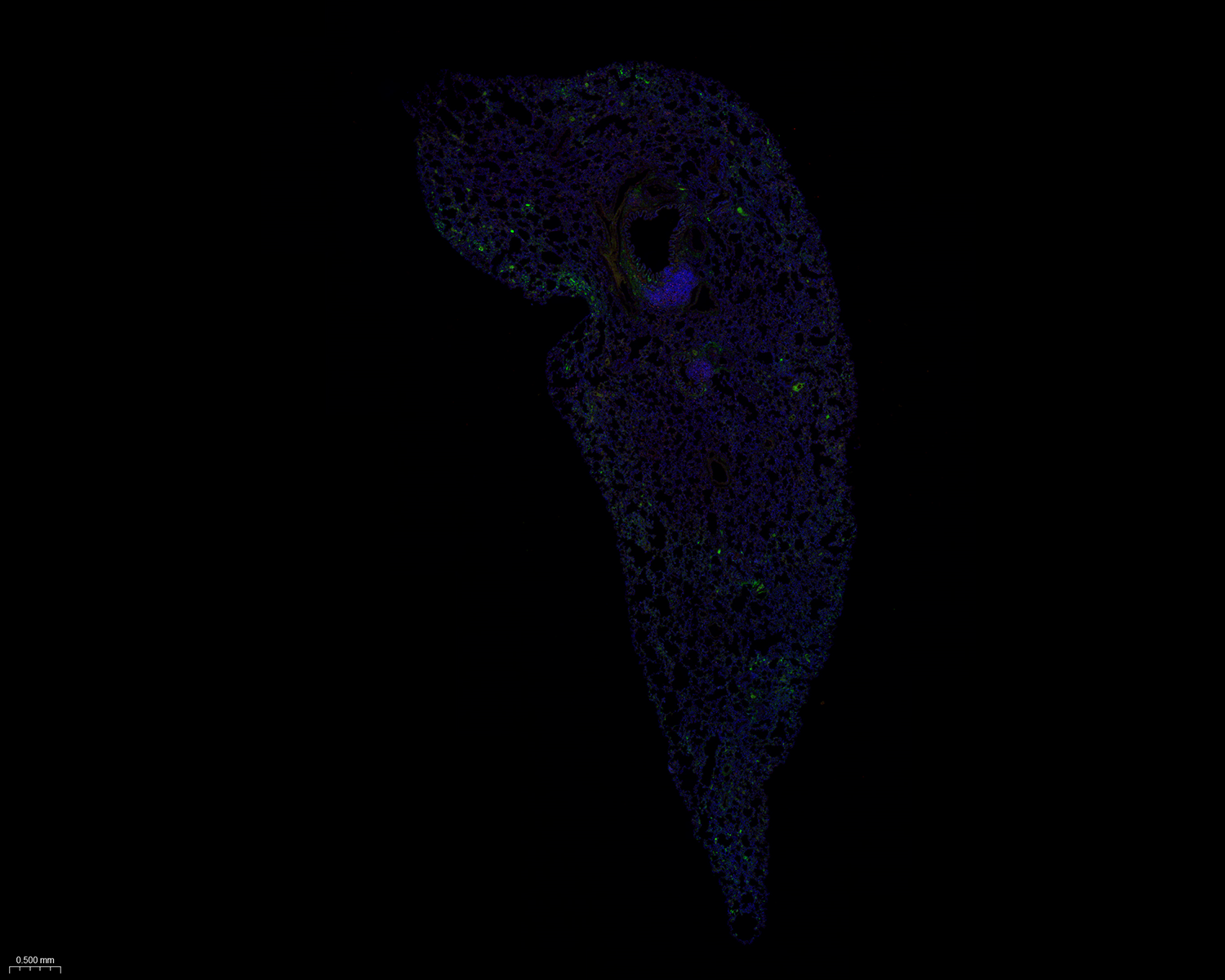

Supplement: Supplementary file 7 [file DataSheet4.zip › Original image of microscope-IF/CK 3.tif]

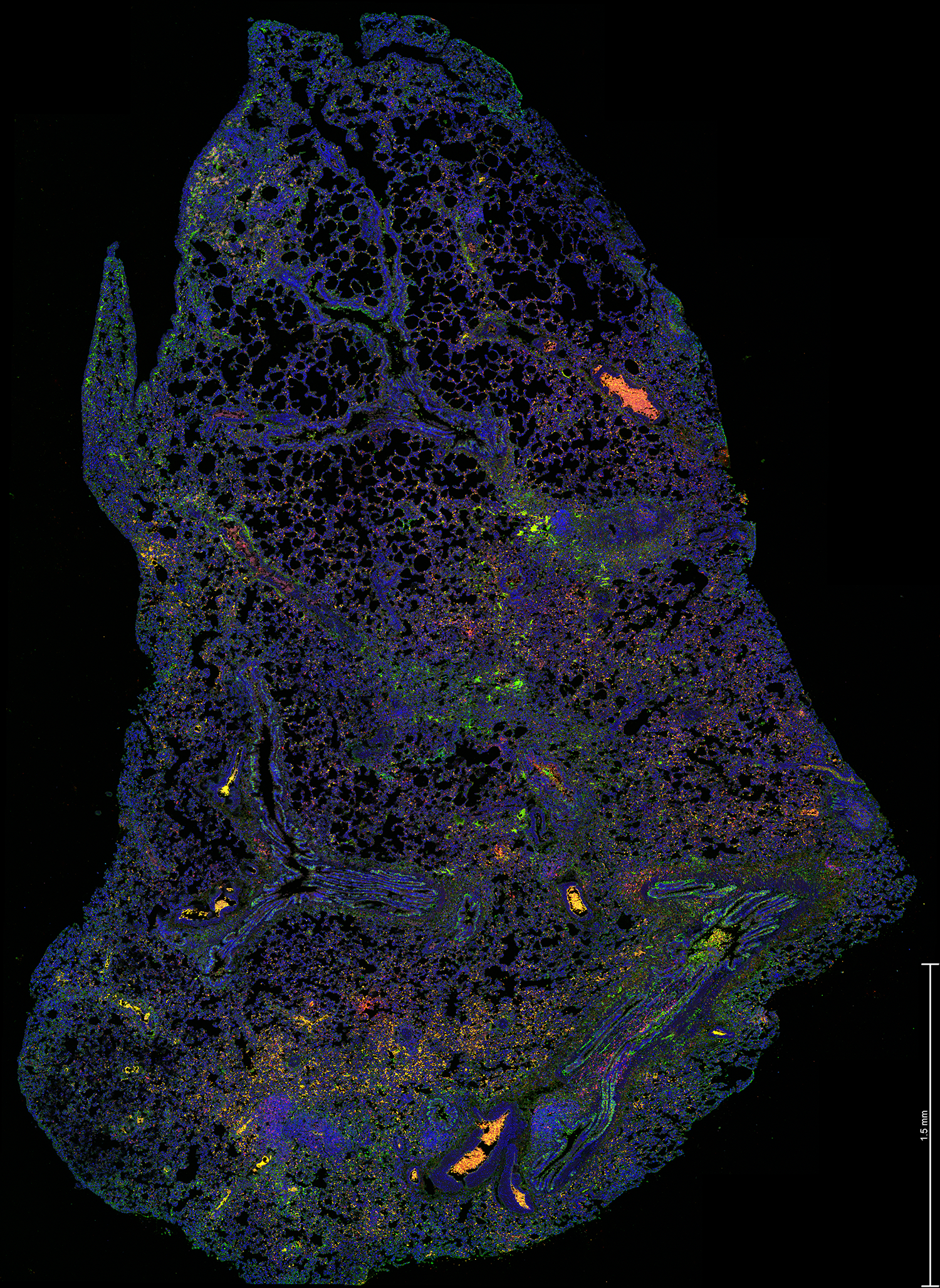

Supplement: Supplementary file 7 [file DataSheet4.zip › Original image of microscope-IF/PAH 1.tif]

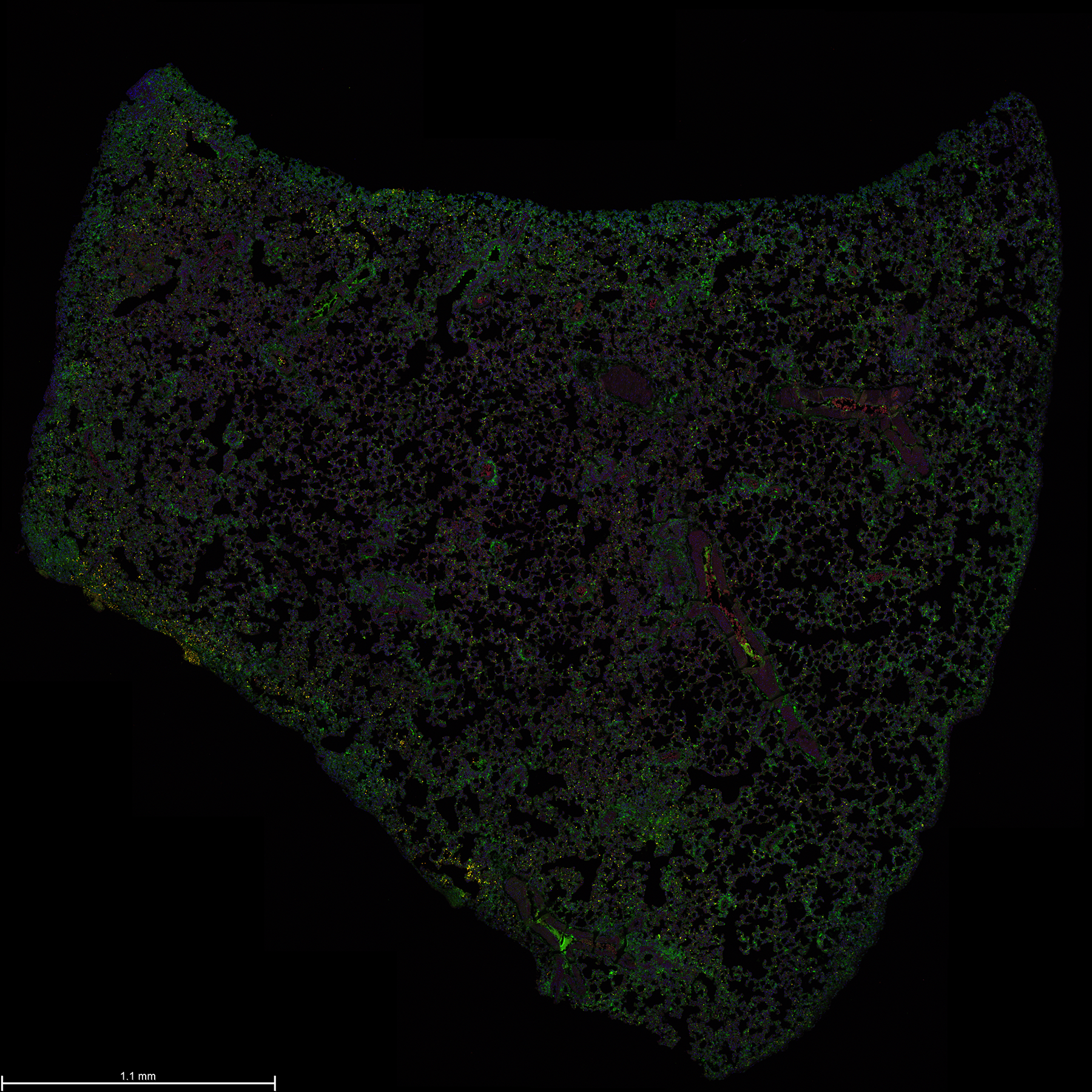

Supplement: Supplementary file 7 [file DataSheet4.zip › Original image of microscope-IF/PAH 2.tif]

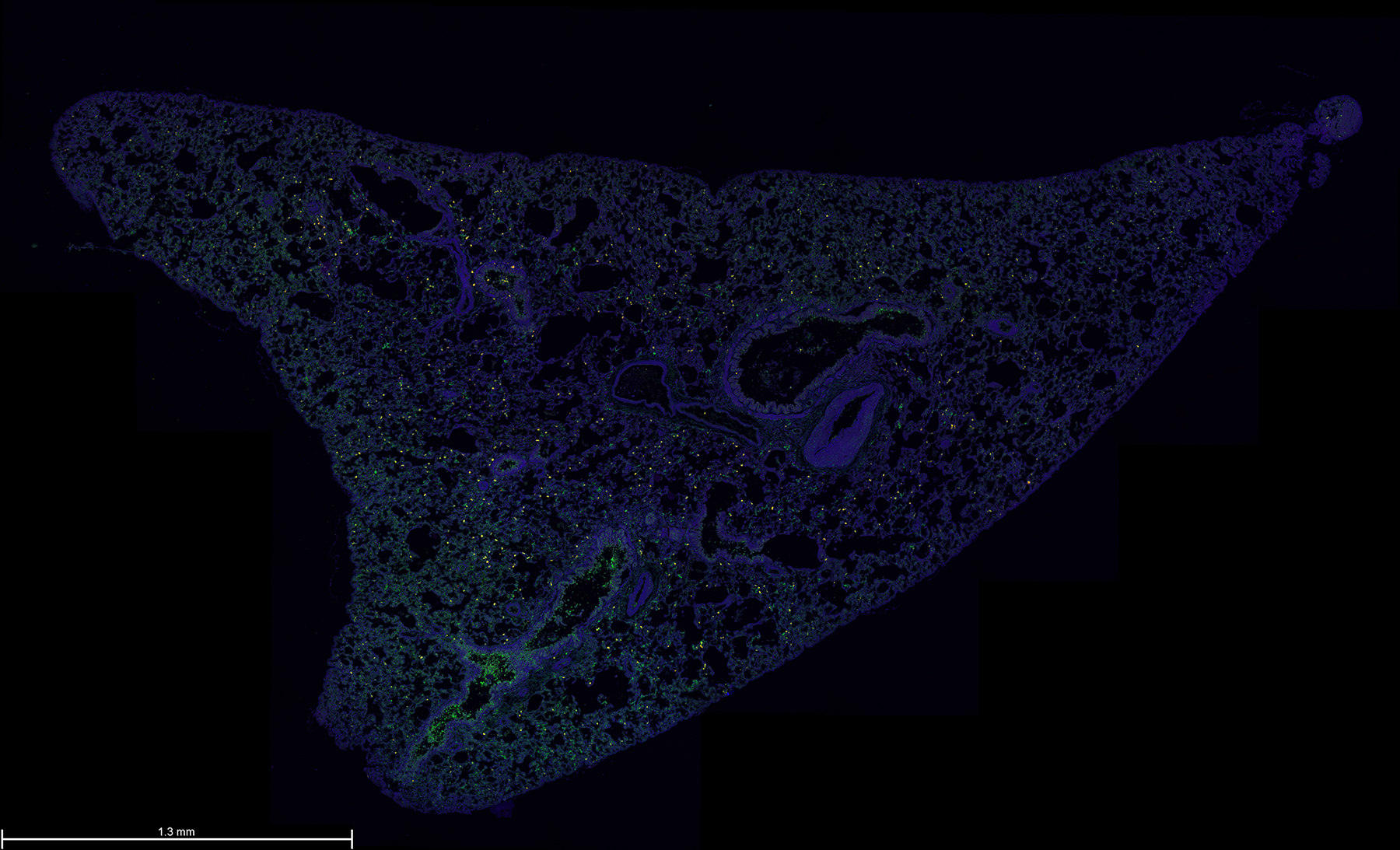

Supplement: Supplementary file 7 [file DataSheet4.zip › Original image of microscope-IF/PAH 3.tif]

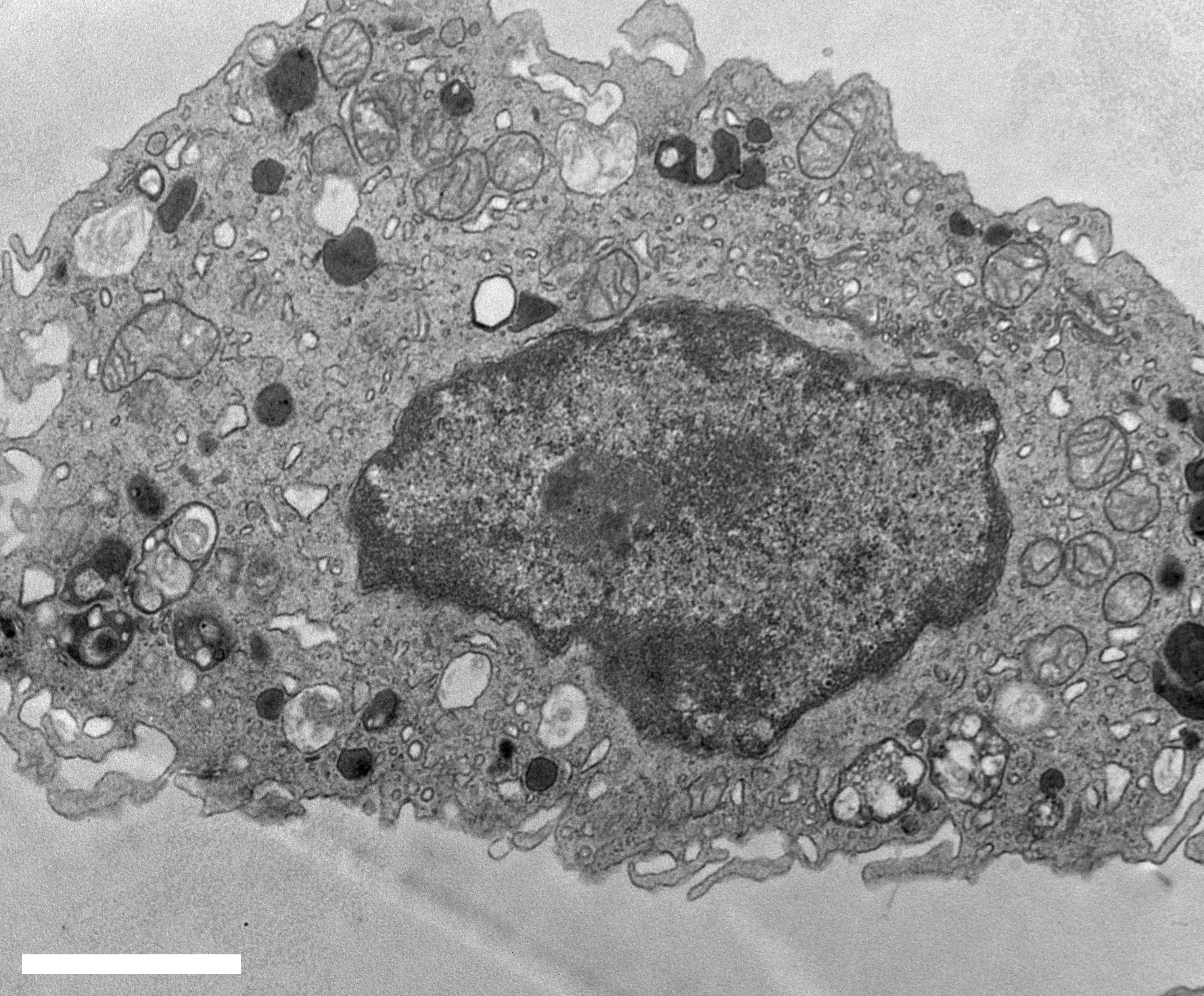

Supplement: Supplementary file 12 [file DataSheet2.zip › Original image of microscope-ET/Fig.CK_Mito(2um).tif]

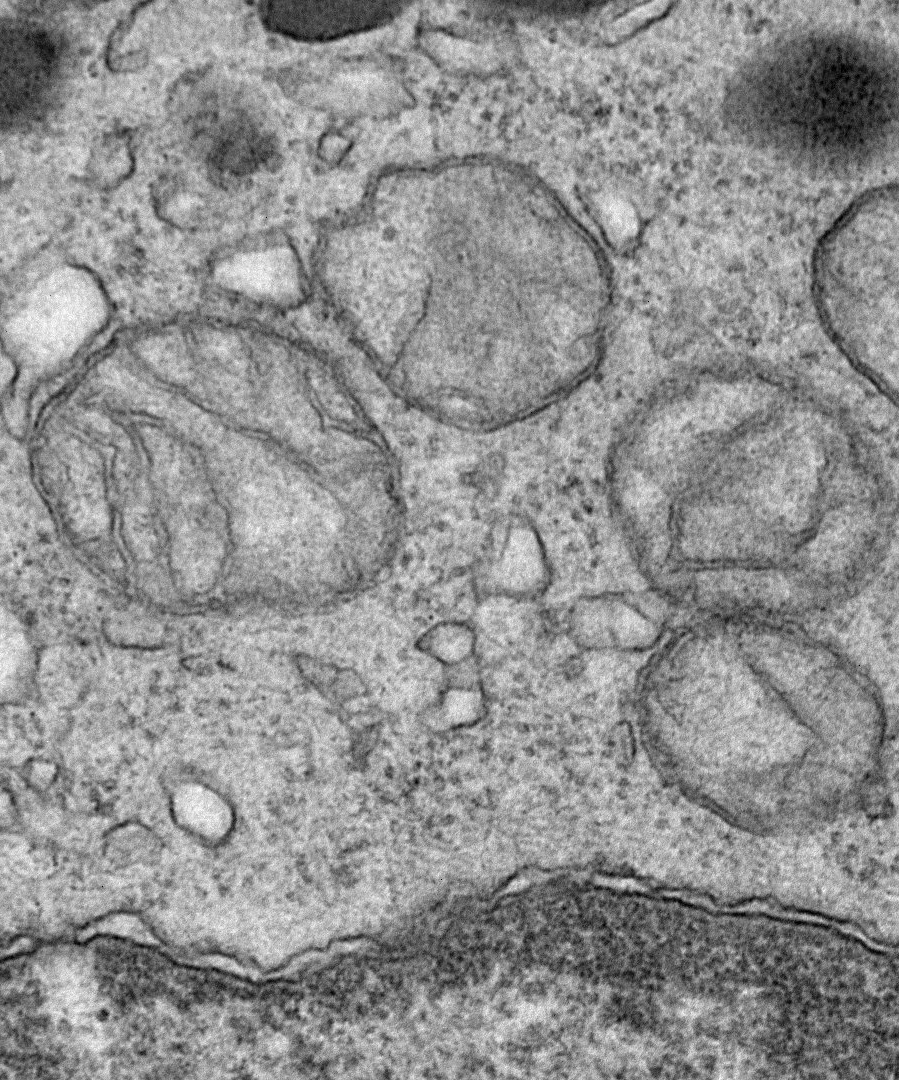

Supplement: Supplementary file 12 [file DataSheet2.zip › Original image of microscope-ET/Fig.CK_Mito_magnify.tif]

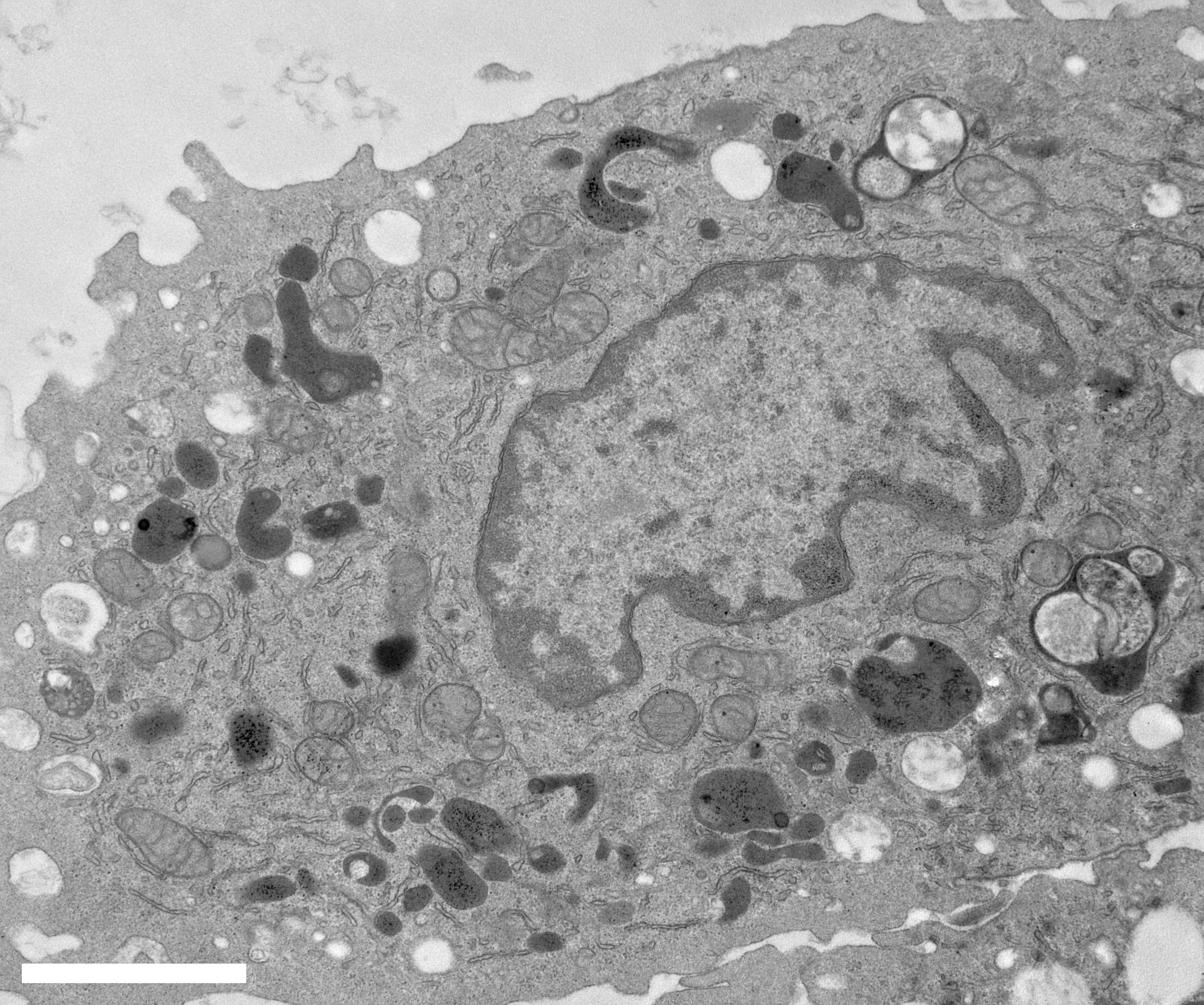

Supplement: Supplementary file 12 [file DataSheet2.zip › Original image of microscope-ET/Fig.PAH_Mito_(2um).tif]

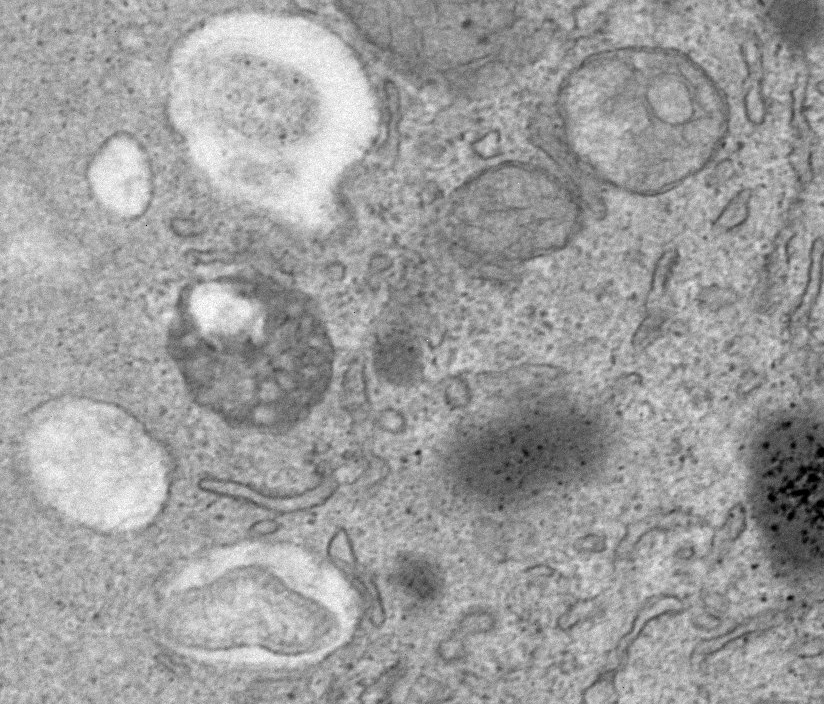

Supplement: Supplementary file 12 [file DataSheet2.zip › Original image of microscope-ET/Fig.PAH_Mitophagy_magnify.tif]
