# Supplementary material for: Mitophagy-associated biomarkers and macrophage involvement in pulmonary arterial hypertension: identification and functional implications
Source: Front Physiol. 2025 Nov 5;16:1673181. doi: 10.3389/fphys.2025.1673181 (PMC12627872; doi:10.3389/fphys.2025.1673181)
Supplement: Supplementary file 5 [file Table1.docx]

Table 1 Primer Sequence

| **Gene** | **Sequence 5’-3’** |
| --- | --- |
| RRAS-F | GTCCTCAGCCCGACATCTCA |
| RRAS-R | GCTGGTCACTTGAGGCTACA |
| BECN1-F | CTTCAATGCGACCTTCCA |
| BECN1-R | TACAACGGCAACTCCTTAG |
| MFN1-F | GCAGCACCAGATAATGCAGC |
| MFN1-R | GCTCTGGTGGAGAAACTGCT |
| HIF1A-F | AAGCAGCAGGAATTGGAACG |
| HIF1A-R | CGTAACTGGTCAGCTGTGGT |
| TAX1BP1-F | TGGATGTAAAGCCAGCAGCA |
| TAX1BP1-R | GCACCATCTGCTCCATCTCT |
| GAPDH-F | AGTCTACTGGCGTCTTCACC |
| GAPDH-R | CCACGATGCCAAAGTTGTCA |
